# Copper Nanoparticle Confinement in P‑Doped Carbon Nitride Templates Enables Efficient Peroxymonosulfate Activation for Alcohol Oxidation

**DOI:** 10.1021/acsomega.6c05865

**Published:** 2026-08-03

**Authors:** Rafael L. Oliveira, Emilia Witkowska-Nery, Sebastian Praetz, Christopher Schlesiger

**Affiliations:** † Institute of Low Temperature and Structure Research of the Polish Academy of Sciences, Wroclaw 50422, Poland; ‡ Institute of Physical Chemistry of the Polish Academy of Sciences, Warsaw 01-224, Poland; § Institute of Physics and Astronomy, 26524 Technical University Berlin, Berlin 10623, Germany

## Abstract

Controlling the particle size and stability under reaction conditions is crucial for increasing catalyst lifetime and producing more sustainable chemical processes. In this work, Cu oxide nanoparticles were confined in three distinct supports: MCF (mesocellular foam) silica, layers of carbon nitride on the surface of mesoporous silica (MCF-CN), or layers of P-doped carbon nitride (MCF-CN-P). The carbon nitride coating was produced using cyanamide, and P-doped carbon nitride was created from a mixture of cyanamide and sodium hypophosphite. The different supports were used to investigate their effects on the distribution, size, and stability of Cu-based particles in the selective oxidation of alcohols, employing peroxymonosulfate (PMS) as the oxidizing agent. Primary and secondary alcohols such as cyclohexanol, benzyl alcohol, and 4-hydroxy-3,5-dimethoxybenzyl alcohol were used as substrates. The synthesized layers of P-doped carbon nitride enhance the conversion of alcohols to their respective ketones or aldehydes, achieving conversions above 80% and selectivity above 90%. Moreover, the stability of copper oxide increases compared with particles deposited on pristine silica.

## Graphical abstract

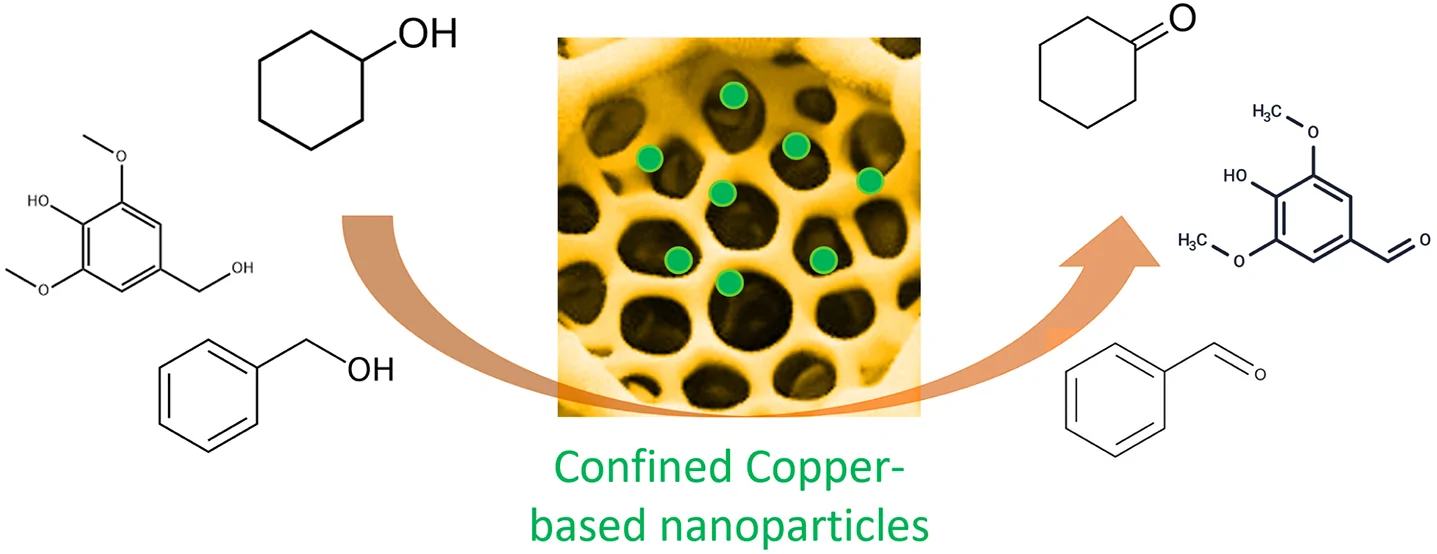

## Introduction

The selective oxidation of functional organic groups, such as hydrocarbons and alcohols, to higher-value compounds, including aldehydes, epoxides, and esters, is a crucial step in the synthesis of pharmaceutical compounds, chemical additives, resins, paints, and polymers.^1–5^ The classical manner for running these oxidation reactions relies on stoichiometric or excess reagents, such as acidified potassium dichromate or permanganate, producing significant waste and, consequently, unsustainable processes from an ecological point of view.^6^

New processes based on heterogeneous catalysts have been reported as alternatives for performing these oxidation reactions using molecular oxygen or liquid peroxide as oxidizing agents. However, these heterogeneous catalysts are commonly based on noble metals such as silver, gold, palladium, and platinum. Thus, these processes face several limitations, including the high cost of catalysts and the challenge of designing reactors to operate under pressurized conditions.^7–17^ Another alternative for producing benzaldehyde is the oxidation of toluene with a Cr-based catalyst at 80–250 °C, a chemical process that involves a toxic metal and high energy consumption.^18^

Attention has been given to the catalytic activation of peroxymonosulfate (PMS) and peroxydisulfate (PSD). Despite the higher cost of PMS and PSD than that of molecular oxygen from air, they are especially interesting due to their stability, which reduces transportation and storage costs and the risk of storage compared to gaseous or explosive liquid-phase oxidants. The catalytic processes using PMS or PSD have been applied to water purification to completely oxidize organic compounds, including pharmaceuticals, pesticides, and dyes.^19,20^ Recently, the catalytic activation of PMS and PSD has also been used to selectively oxidize alcohols and hydrocarbons with various catalysts.^21–27^ For example, Zhao et al. immobilized Mn single atoms on N-doped graphene and used the solid catalyst to selectively oxidize alcohols. The synthesized catalyst shows superior conversion and selectivity for aldehydes compared to homogeneous Mn ions and oxide particles. The authors attributed this performance to a synergistic effect between Mn and N-doped carbon.^28^

The use of earth-abundant metals (Fe, Ni, Co, and Cu) to develop solid-state catalysts has been an exciting research topic in catalysis over the past decade. The disadvantage of these catalysts is that they usually yield materials with lower performance than those of noble metals. Thus, harsh conditions, such as higher pressure (when gases are used as the oxidizing agent), temperature, and prolonged reaction times, are commonly required. Thus, controlling the metal particle size and metal–support interaction on these catalysts is a key aspect of producing desired materials with higher catalytic activity.^29–33^ Cu-based catalysts have been identified as among the most promising metals for selective oxidation using O_2_ or TEMPO as oxidizing agents; however, most reports focus on immobilized complexes, homogeneous catalysts, or combinations of copper with noble metals.^34–38^

Graphite carbon nitride (g-C_3_N_4_) is a two-dimensional layer material that has been extensively explored as a catalyst support for a variety of metal nanoparticles. Its N-rich chemical environment drives a strong coupling between metal–support interactions and particle size control.^39–43^ Moreover, it possesses good chemical stability and is relatively low-cost. Carbon nitride has also been doped with sulfur, boron, and phosphorus to change its physical and chemical properties.^44–46^ Phosphorus doping is known to enhance the electronic properties of C_3_N_4_, thereby improving electrical conductivity.^47–49^

The disadvantages of using two-dimensional materials as catalysts include the ease with which deposited metal particles can migrate on the support surface, leading to rapid deactivation due to metal particle growth.^50,51^ Thus, synthesizing porous carbon nitride-based materials with a porous structure in which metal nanoparticles can be confined is highly desirable.^52,53^ A standard methodology for producing these structures is a hard-template technique, in which a preformed template is used to synthesize them.^44,54–57^ These templates are commonly oxide nanoparticles or well-ordered mesoporous silica, such as SBA-15 and mesocellular foam silica (MCF).^58–60^

Herein, three distinct supports were synthesized: an MCF, carbon nitride layers deposited on the MCF surface (MCF-CN), and P-doped carbon nitride layers formed on the MCF (MCF-CN-P), producing highly porous catalytic supports. The copper oxide nanoparticles were confined in these structures, allowing comparison of the support’s effect on metal particle distribution, size, chemical environment, and stability, while also exploring a possible synergistic effect between the metal oxide particles and the support. These three distinct Cu-based solids were used as catalysts for the selective oxidation of cyclohexanol, benzyl alcohol, and 4-hydroxy-3,5-dimethoxybenzyl alcohol (a biomass-derived compound) with PMS as the oxidant.

## Results and Discussion

Figure 1A illustrates the synthesis scheme for the composites. Layers of carbon nitride or P-doped carbon nitride were synthesized by infiltrating a cyanamide solution or a mixture of cyanamide and sodium hypophosphite into the porous structure of MCF, followed by thermal treatment under N_2_ atmosphere, resulting in MCF-CN (MCF with layers of carbon nitride) and MCF-CN-P (MCF with layers of P-doped carbon nitride). MCF was selected as a template due to its large, interconnected porous structure (Figures 1B and S1), which reduces pore blockage and mass-transfer limitations. Moreover, TEM images of MCF-CN and MCF-CN-P are shown in Figure S1B–C.

**Figure 1:**
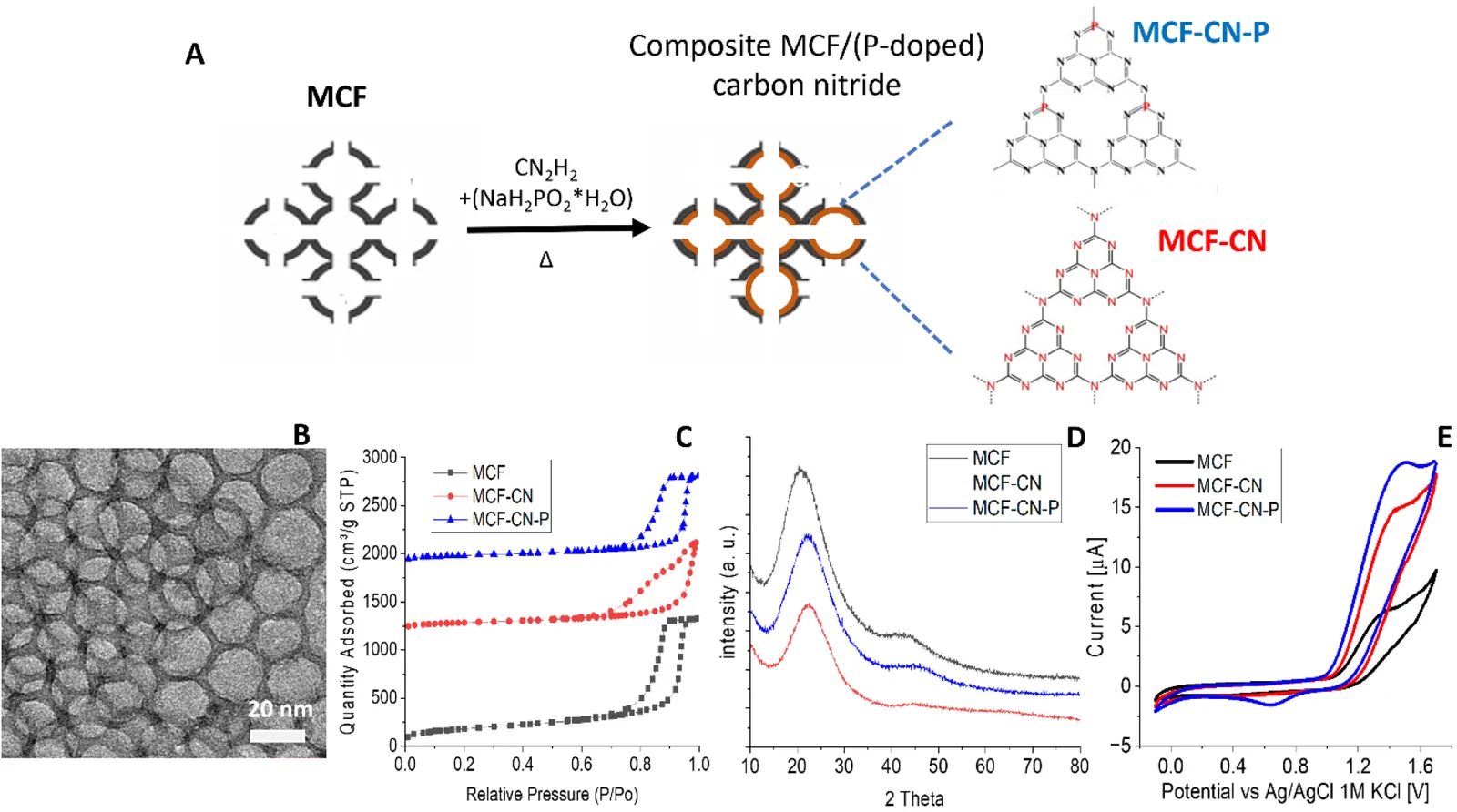
(A) Schematic procedure for synthesizing composite materials; (B) TEM image of MCF silica; (C) N_2_ physisorption isotherms at −196.5 °C; the isotherms of MCF-CN and MCF-CN-P were offset by 1200 and 1900; (D) XRD pattern of pristine silica and the composites; (E) Cyclic voltammetry in 10 mM PMS in acetonitrile, scan rate 20 mV/s.

N_2_ physisorption isotherms at −196.15 °C of pristine and modified materials are displayed in Figure 1C. The isotherms of MCF and MCF-CN-P can be classified as type IV following the IUPAC classification with a hysteresis H1; however, MCF-CN presented a different type of hysteresis H2b, which indicates a broader range of size in pore restriction or pore entrances, suggesting that a P-doped carbon nitride has a thinner layer than carbon nitride. Introducing these layers onto MCF causes a loss of surface area, pore volume, and pore size, as expected (Table S1). TG analysis was used to determine the loading of organic matter on the silica surfaces. Figure S2 shows the thermograms of MCF-CN and MCF-CN-P. The mass loss after thermal treatment was around 20 wt %.

XRD patterns of the synthesized materials are shown in Figure 1D, with two broad peaks at approximately 21° and 44°, characteristic of amorphous silica. Carbon nitride-derived materials also showed a peak around 25 °, which might overlap with an amorphous silica peak, consistent with the work of Zhu et al.,^55^ who synthesized carbon nitride in SBA-15 at 500 °C. The authors were unable to obtain the diffractogram of the carbon nitride structure. No porous carbon nitride was synthesized; the XRD pattern is shown in Figure S3, with two major peaks at approximately 13.0° (low-intensity) and 26.9°, which correspond to the intra- and interlayer distances of the graphitic carbon nitride structure, respectively.^54,61^

The FTIR spectra of the synthesized porous materials are shown in Figure S4. The bands at around 431, 794, and 1065 cm^–1^ can be attributed to asymmetric stretching or bending vibrations of Si–O–Si bonds. A small peak on the MCF spectra at about 1628 cm^–1^ can be assigned to adsorbed water.^50,62,62^ The MCF-CN and MCF-CN-P exhibit peaks in the range of 1275–1748 cm^-1^, typically assigned to stretching vibrations of the heptazine units. An increase in absorbance intensity at around 1640 cm^–1^ in MCF-CN and MCF-CN-P can be assigned to conjugated C–N stretching. Another band centered around 1447 and 1574 cm^–1^ is assigned to the stretching modes of the tri-s-triazine ring, and the band at 1324 cm^-1^ is assigned to secondary bridging N.^54,63^

Cyclic voltammetry (CV) in acetonitrile solution containing PMS was used to characterize the electrochemical behavior of the materials. The comparative CV (Figure 1E) demonstrated that the introduction of carbon nitride or P-doped carbon nitride significantly increased the oxidation peak at around 1.4 V as compared to MCF. The addition of a P dopant also enhanced the electrochemical properties of this support, with an even higher oxidation current at around 1.4 V and the appearance of a reduction peak at around 0.55 V, thereby yielding supports with a better ability to store charge, in line with previous reports.^47,48^

The optical properties of porous composites were also examined using UV–vis spectroscopy (Figure S5), MCF-CN and MCF-CN-P presented standard semiconductor absorption spectra of g-C_3_N_4_-derived materials with a broad absorbance between 200 and 400 nm; however, MCF-CN-P presented a larger absorbance in the ultraviolet range below 230 nm and above 385 nm, and a smaller absorbance in the range of UV light around 230–379 nm. The redshift is also consistent with Fang et al., who attributed it to a color change in the obtained powder from light (carbon nitride) to a darker yellow (doped carbon nitride).^64^ This blueshift might be associated with the introduction of phosphorus atoms, interactions with the lone-pair electrons of the aromatic nitrogen atoms, and the size effect of the deposited nanosheet layer.^65,66^

SEM images of pristine silica and composites are shown in Figure S6; all samples exhibited a uniform, spherical morphology, and the deposition of the carbon nitride layer did not cause any drastic changes in the silica structure. Carbon, nitrogen, and phosphorus distributions across the silica surface of MCF-CN-P were determined by energy-dispersive X-ray spectroscopy (EDS); see Figure 2 (top frame). The scanning elements show a homogeneous distribution over the five MCF particles presented in the image. HAADF-STEM combined with EDS chemical mapping of an MCF-CN pore is shown in Figure 2 (bottom frame); silicon, oxygen, and carbon were scanned. The Si and O mapping demonstrated that the wall of MCF is composed of Si–O–Si, as expected. C mapping also suggests the formation of a layer of carbon nitride on MCF’s walls.

**Figure 2:**
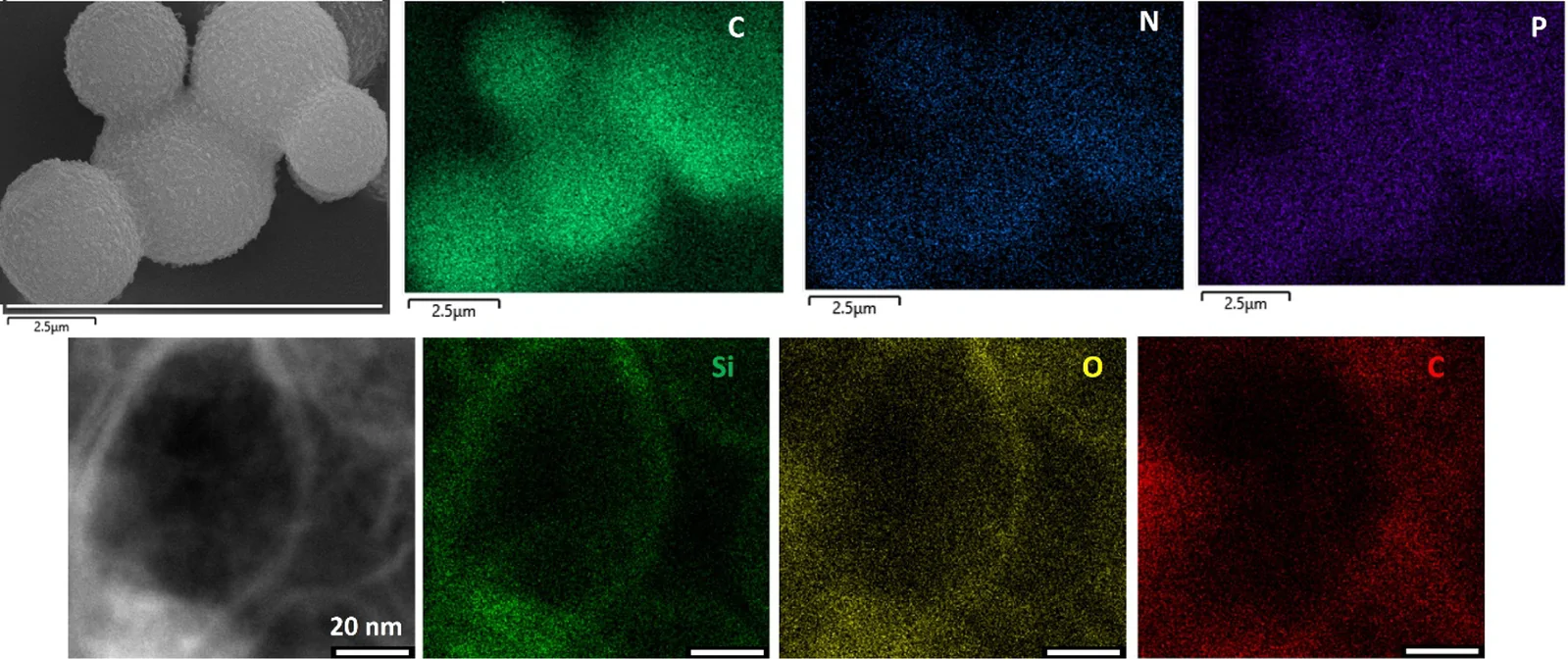
SEM image of MCF-CN-P combined with EDS chemical mapping of C, N, and P (top frame); HAADF-STEM EDS chemical mapping of Si, O, and C of a pore of MCF-CN (bottom frame).

X-ray photoemission spectroscopy (XPS) was also used to confirm the formation of carbon nitride and P-doped carbon nitride on the silica structure. Figure 3 shows the C 1s, N 1s, and P 2p high-resolution spectra of MCF-CN and MCF-CN-P. The spectra of C 1s (Figure 3A) could be deconvoluted into five peaks centered at about 284.9, 285.5, 286.7, 288.0, and 289.4 eV. The peak at 284.9 eV corresponds to standard reference carbon (graphitic carbon is commonly observed in the XPS spectra of carbon nitrides).^67,68^ The terminal sp carbon (carbon–nitrogen triple bond) is assigned to the peak at 285.5 eV, and 286.7 eV is assigned to a C-NH_2_ bond. The peak at 288.0 eV corresponds to the sp^2^-bonded carbon ring (N–C = N) typical of g-C3N4.^69^ Finally, the peak at 289.4 eV is attributed to C–O, which may be related to surface oxidation or direct interaction with silanol groups on MCF. The peaks of MCF-CN and MCF-CN-P differ only slightly, with Csp showing higher intensity in the MCF-CN sample and C-NH_2_ lower. The quantified values are shown in Table S2.

**Figure 3:**
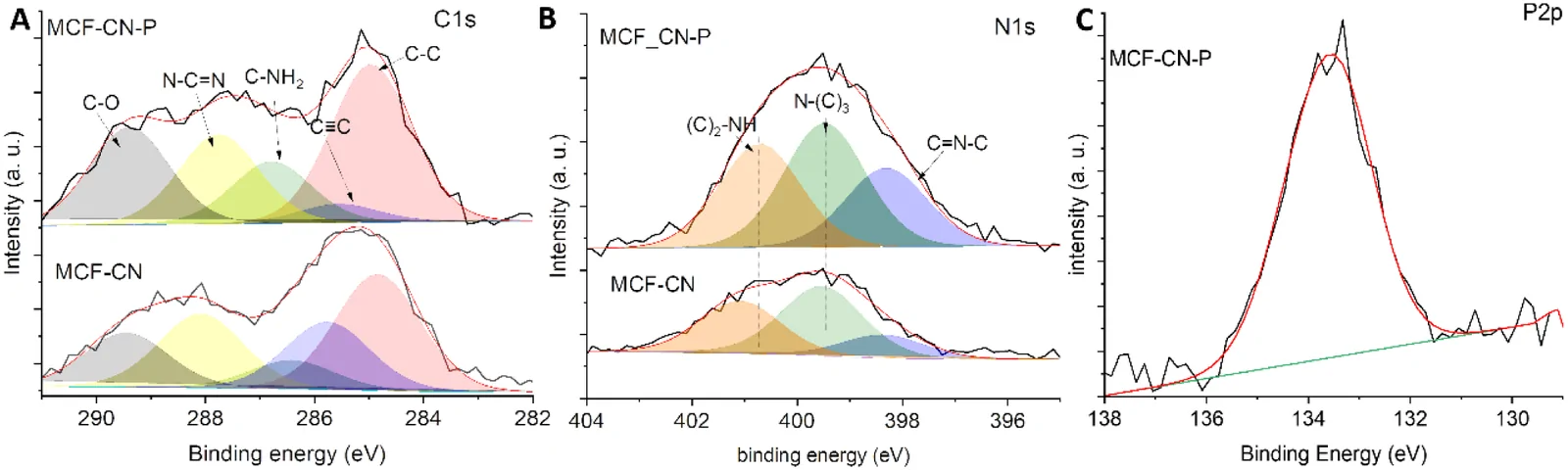
High-resolution XPS of the composites (A) C 1s, (B) N 1s and (C) P 2p.

Figure 3B shows the high-resolution XPS N 1s spectra. The spectra could be deconvoluted into three peaks at around 398.3, 399.5, and 400.7 eV. The peak at 398.3 eV can be attributed to sp^2^-hybridized nitrogen in triazine rings (N–C = N); at 399.5 eV, it is commonly associated with tertiary nitrogen N-(C)_3_ groups; and, finally, at 400.7 eV, it is associated with amino-carrying hydrogen species (NH_2_).^69,70^ Furthermore, the shifts to lower binding energy observed in the N 1s spectra of MCF-CN-P have been reported elsewhere, suggesting that the introduction of P atoms disrupts the basic structure of carbon nitride (formation of N defects) and, as a consequence, causes a change in the electronic structure.^65–73^ The distribution of N-bond types is shown in Table S3.

XPS O 1s is shown in Figure S7; the spectra indicate three chemical environments: Si–O–Si, a major peak placed at 533.3 eV, and two less intense peaks located at 532.3 eV, which correspond to the (Si–O–C) groups, indicating a chemical interaction between the formed layers of carbon nitride and the template. Finally, a peak at 534.4 eV was assigned to silanol groups (Si–OH).^50,74,75^ Figure S8 displays the high-resolution Si 2p XPS of the MCF-CN and MCF-CN-P. The spectra were deconvoluted to a single peak at 104 eV, indicating that Si–O–Si is the dominant chemical state of silicon. The high-resolution P 2p spectrum shows a single peak at 133.5 eV, characteristic of a P–N chemical bond. A single P 2p has been associated with P atoms in an identical chemical environment in a heptazine ring system.^48,76^

The deposition of Cu species on the porous support was carried out by incipient wetness impregnation, followed by thermal treatment under an Ar atmosphere. XRD patterns are shown in Figure 4A; MCF-CN-Cu and MCF-CN–P-Cu do not display any peak related to Cu species, suggesting the formation of small clusters dispersed throughout the support or single Cu atoms. On the other hand, MCF-Cu presents peaks, indicating the formation of much larger particles. Two sharp, low-intensity peaks are centered at 35.6° and 38.8° and are associated with the lattice planes −111 and 111, respectively, of a monoclinic phase of CuO (space group C12/c1; JCPDS card #41–0254), as shown in Figure S9.^77–79^

**Figure 4:**
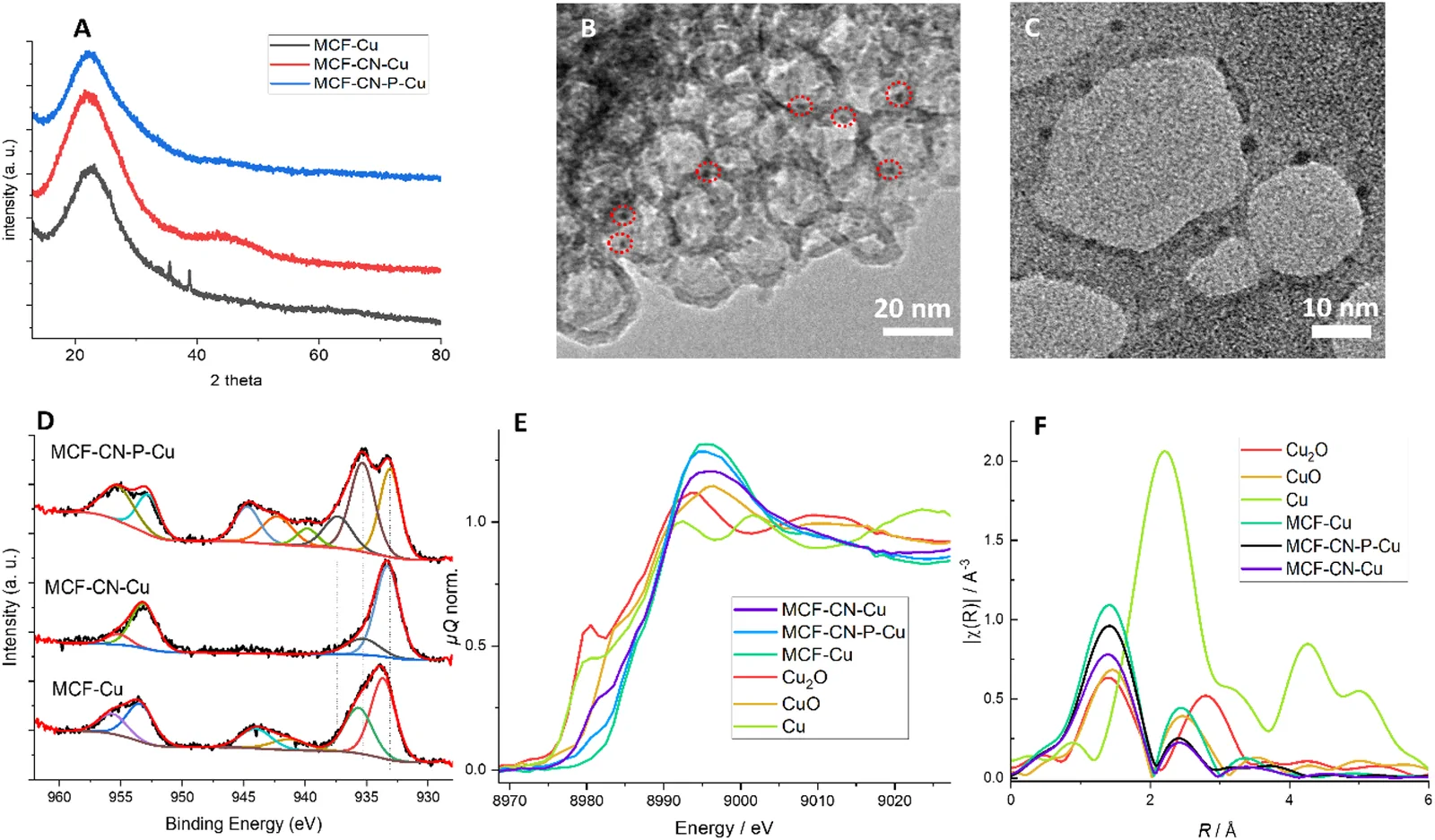
(A) XRD of MCF-Cu, MCF-CN-Cu, MCF-CN–P-Cu; (B and C) TEM images of MCF-CN–P-Cu; (D) XPS high-resolution of Cu 2p; (E) normalized Cu K-edge XANES spectra; and (F) normalized FT-EXAFS of the synthesized materials and references.

Figure 4B and C display TEM images of MCF-CN–P-Cu, with small metal oxide clusters (dark spots) highlighted in red. Moreover, Figure 4C shows a high-resolution image in which the metal-oxide clusters are confined within the support’s mesopores. The micrographs of MCF-CN-Cu are shown in Figure S10, where small metal-oxide clusters are visible and confined into the porous structure of the support. The TEM of MCF-Cu is shown in Figure S11, which presents dark-field and bright-field images revealing much larger particles with a broad size distribution ranging from 2 to 40 nm, consistent with the XRD analysis.

Figure 4D shows the high-resolution XPS Cu 2p spectra. The supports strongly influence the Cu spectrum. All samples exhibit two major peaks at around 934 and 953 eV, corresponding to the Cu 2p_3/2_ and Cu 2p_1/2_ core levels, and a third peak at 943 eV, a satellite commonly observed in metal oxides.^33,39,80^ The band of Cu 2p_3/2_ could be deconvoluted into two peaks centered at 933 and 935.4 eV, corresponding to Cu^2+^ in the copper oxide structure (CuO) for MCF-Cu and MCF-CN-Cu. The spectrum of MCF-CN–P-Cu shows an additional deconvoluted peak at 937.4 eV, which may be associated with a highly oxidized state of Cu atoms or Cu–OH.^81,82^ The deconvoluted peaks at 932.9 and 935.4 eV of MCF-CN-Cu and MCF-CN–P-Cu suffer a shift to lower energies; this shift is slightly bigger for MCF-CN–P-Cu, which implies an electron transfer from the carbon nitride or P-doped carbon nitride to the metal-oxide cluster particle via the Cu–N_*x*_ or Cu–P bond, which gives the Cu species an oxidative state between +2 and +1.^83,84^

The normalized Cu K-edge X-ray absorption near-edge structure (XANES) spectra of the synthesized materials and the references (Cu foil, Cu2O, and CuO) are shown in Figure 4E. The absorption edges of Cu foil, Cu_2_O (Cu^+^), and CuO (Cu^2+^) are centered at approximately 8992.2, 8993.4, and 8996.1 eV, respectively. The spectra of the supported copper species show a broader band between 8991 and 9000 eV, centered at 8994.8 eV for MCF-CN-Cu and MCF-CN–P-Cu, and slightly higher for MCF-Cu at 8995.4 eV. These absorption energies would be between those of CuO and Cu_2_O species. Due to the broadness of these peaks, some authors indicate an oxidative stage between +1 and +2 for the Cu species,^85,86^ in line with XPS analysis.

Moreover, a strong shoulder (prepeak) is observed for MCF-CN-Cu, which is not the case for MCF-Cu, and only a slight shoulder (prepeak) is observed for MCF-CN–P-Cu. Prepeaks arise mainly from electronic transitions (like 1s → 3d), which are typically weak unless enhanced by 3d–4p hybridization in low-symmetry environments. The intensity and position are influenced by local symmetry, crystal-field effects, and oxidation state, all of which affect orbital mixing and the availability of unoccupied states.

Fourier transform extended X-ray absorption fine structure (FT-EXAFS) analyses were used to address the chemical environment of Cu atoms in these solids. Figure 4F shows the spectra and radial distribution functions of the synthesized materials and references (Cu foil, Cu_2_O, and CuO). The deposited Cu oxide on MCF-based materials shows a slight shift in the Cu–O bond length in the first shell, which is commonly associated with the smaller size of nanometric particles relative to bulk references.^87,88^ The same shift in Cu–O bond length was observed for the second and third shells; however, it was more pronounced for MCF-CN-Cu and MCF-CN–P-Cu, suggesting a chemical interaction between Cu-oxide clusters and N or P atoms on the support. Moreover, MCF-Cu, particularly the first and second peaks in the FT-EXAFS, shows higher values than those of MCF-CN-Cu and MCF-CN–P-Cu, further corroborating a different coordination environment.

### Catalyst Tests

The catalytic performance of the synthesized materials was evaluated for the selective oxidation of cyclohexanol, benzyl alcohol, and a more complex substrate, 4-hydroxy-3,5-dimethoxybenzyl alcohol, via activation of peroxymonosulfate (PMS). The selection of these alcohols was based on the numerous applications of the corresponding ketones and aldehydes in the chemical industry, which together account for a global market worth hundreds of millions of euros.

Figure 5 shows the conversion and selectivity for the corresponding ketone and aldehyde using acetonitrile as solvent; the yield values are shown in Table S4. The blank experiment was performed without adding the catalyst to the reaction mixture. The conversion of the substrate in the blank reaction was less than 10%, with a selectivity of around 80% for all cases. The supports without the copper-based nanoparticles were also tested. The pristine MCF has not shown any improvement in catalytic activity; however, MCF-CN and MCF-CN-P have already demonstrated potential as catalysts, with conversions of 31% and 45%, respectively, for the selective oxidation of cyclohexanol (Figure S12). Graphitic carbon nitride was also used as a support to investigate the influence of porosity in MCF-CN and MCF-P. The nonporous support yields a conversion of 12%, much lower than that of MCF-CN and MCF-CN-P. The introduction of Cu onto MCF-CN and MCF-CN-P led to a substantial increase in conversion and a slight increase in selectivity. Moreover, 4-hydroxy-3,5-dimethoxybenzyl alcohol is more resistant to oxidation, requiring a longer reaction time.

**Figure 5:**
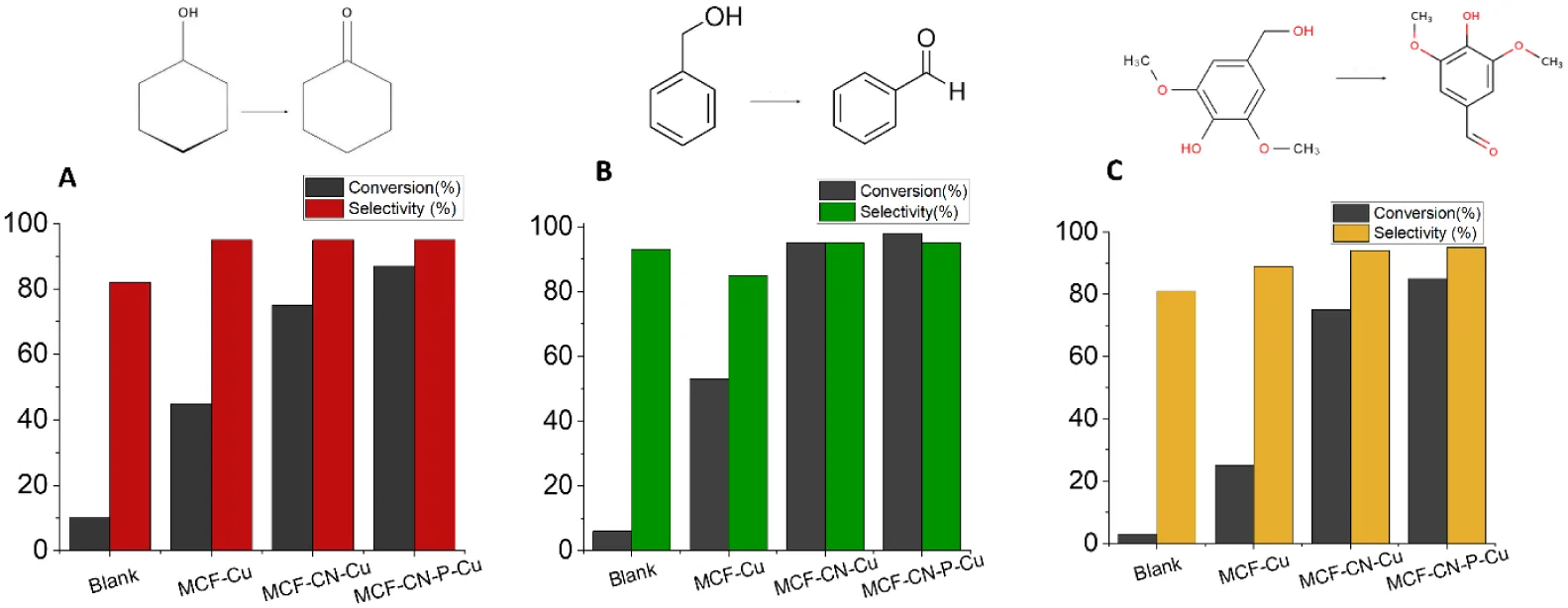
Conversion and selectivity in the selective oxidation of (A) cyclohexanol, (B) benzyl alcohol, and (C) 4-hydroxy-3,5-dimethoxybenzyl alcohol. Conditions: 0.5 mmol of the desired alcohol, 0.55 mmol of PMS, 5 mL of acetonitrile, 15 mg of Cu-catalyst, 2 h (for cyclohexanol and benzyl alcohol) or 6 h (for 4-hydroxy-3,5-dimethoxybenzyl alcohol) at 50 °C.

A linear primary alcohol (1-octanol) was also explored as a substrate. It has been reported that linear alcohols are more resistant to oxidation; moreover, linear aldehydes are less stable than aromatic compounds.^88,89^ Figure S13 shows the catalytic activity and selectivity for the selective oxidation of 1-octanol. Once again, MCF-CN–P-Cu demonstrates the best catalytic activity among the other catalysts. Moderate selectivity of 1-octanal was observed (major product), with the formation of carboxylic acid as a byproduct.

The choice of solvent plays an essential role in the reaction. Figure S14A shows the conversion of cyclohexanol in various polar and apolar solvents, with conversions ranging from 10% to 90%. Some authors reported that using a mixture of water and acetonitrile as a solvent could achieve considerably high conversions and selectivities.^21,23,24,90^ However, in our hands, this mixture of solvents caused a rapid conversion of cyclohexanol into secondary products, culminating in its complete decomposition, suggesting the formation of excess radicals that drive the reaction mechanism. The catalytic decomposition of persulfate in water is well documented and widely used to remove emerging contaminants.^91,92^ Figure 6A shows that increasing the water content in acetonitrile from 10% to 100% V/V results in a drastic decrease in cyclohexanone selectivity, leading to its complete decomposition at water contents above 50% V/V. Moreover, we also explored the loading (catalyst mass) of MCF-CN–P-Cu. Figure S14B shows that the best catalyst performance was achieved at 15 mg, with conversion and selectivity above 90%. With higher catalyst loading, we observed a slight decrease in selectivity.

**Figure 6:**
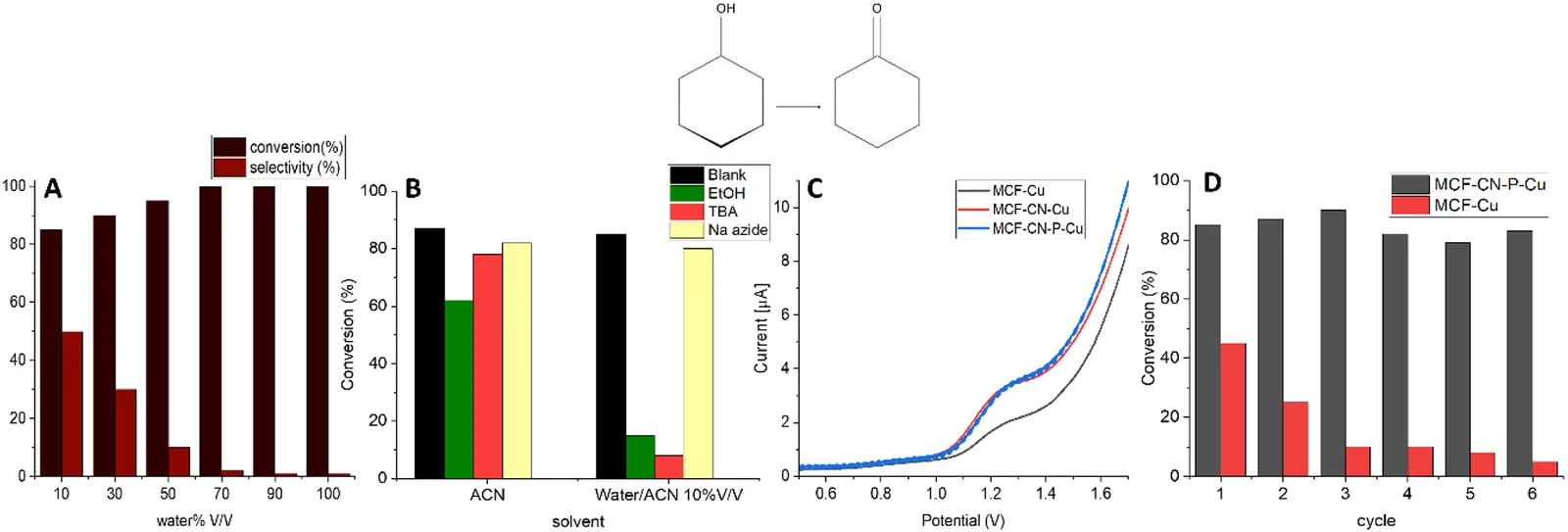
(A) Influence of water in the selective oxidation of cyclohexanol using MCF-CN–P-Cu, conditions: 0.5 mmol of cyclohexanol, 0.4 mmol of PMS, 5 mL of solvent, 15 mg of MCF-CN–P-Cu, 2 h, 50 °C. (B) Quenching test with ethanol, tert-butanol, and sodium azide, conditions: 0.5 mmol of cyclohexanol, 0.4 mmol of PMS, 4 mL of ACN, 1 mL of EtOH, or 1 mL of TBA, 2 h, 50 °C (C) Linear-sweep voltammograms of the Cu-based catalysts, (D) recyclability of MCF-CN–P-Cu and MCF-Cu in the selective oxidation of cyclohexanol, conditions: 0.5 mmol of cyclohexanol, 0.4 mmol of PMS, 5 mL of solvent, 15 mg of MCF-CN–P-Cu, 2 h, 50 °C.

Quenching tests can elucidate the oxidizing species and the mechanisms underlying these selective oxidations. Ethanol (EtOH) is a known agent to quench OH^.–^ and SO_4_^.–^ radicals with high reaction velocity and tert-butanol (TBA) is also commonly used to track the OH^.–^ and is known to be less efficient to track SO4^.–^.^93,94^ Figure 6B shows two distinct experimental sets, in which ACN was used as a solvent or as a 10% water/ACN (V/V) mixture. When ACN was used, the introduction of tert-butanol led to a slight decrease in conversion (around 10%). However, the addition of ethanol drove a conversion suppression of about 20%, demonstrating that the radical SO_4_^.–^ can be responsible for a small portion of the conversion, and a nonradical path might play an essential role in this catalytic system.

It is known that Cu (II) can activate PMS via a radical path and turn into Cu­(III) (the chemical equations are shown in Supporting Information, eqs 1–3). The catalyst is regenerated via reduction of Cu (III). The catalyst support can play an essential role in this system, transferring electrons or facilitating the reduction of metal species. The cyclic voltammetric analyses of the supports (Figure 1E) show that MCF-CN-P exhibits a greater tendency for electron transfer (the lowest cathodic peak potential, Epc) than MCF and MCF-CN.

In the catalytic test using 10% water in acetonitrile (V/V; Figure 6B), the addition of ethanol or tert-butanol leads to a dramatic decrease in conversion. Tert-butanol causes a conversion suppression even greater than that of ethanol, suggesting that adding water might promote the formation of the OH^.–^ radical, which is responsible for the overoxidation of cyclohexanol. PMS is used as an oxidizing agent to remove organic compounds from water in advanced oxidative processes via radical formation.^95^

Another possible mechanism for the selective oxidation of cyclohexanol is the generation of singlet oxygen (^1^O_2_), which may arise from the decomposition of PMS. Quenching tests with ethanol and tert-butanol are inefficient at detecting singlet oxygen. The literature shows uncertainties about the protocol for establishing a methodology to determine a nonradical path. Sodium azide is one of the most common chemicals used as a quenching agent for singlet oxygen. Herein, adding sodium azide (5 equiv) to the reaction mixture shows a slight reduction of cyclohexanol conversion, indicating a small or no influence of singlet oxygen as an oxidizing agent in this reaction’s mechanism.

A direct electron-transfer mechanism has also been reported as the primary mechanism for some oxidation processes in which PMS is used as the oxidizing agent, in combination with conductive materials such as carbonaceous materials.^93,96^ Electrochemical analyses commonly identify this mechanism. Figures 6C and S15 show linear sweep voltammetry measurements in the presence of cyclohexanol or benzyl alcohol and PMS, respectively. The carbon nitride or P-doped carbon nitride layer exhibits better electron-transfer ability than the pristine MCF; moreover, P-doping also enhances efficiency. The efficiency of the catalysts reported herein was compared with other previously reported materials, demonstrating the best catalytic activity among them (Table S5).

A proposed mechanism is that carbon nitride, or P-doped carbon nitride, can act as a bridge between the substrate (electron donor) and the PMS (electron acceptor) in the absence of radicals. It is known that N-species can act as a linker to slightly acidic alcohols, and the oxygen species on silica could serve as an adsorption linker for PMS. At the same time, the metal-oxide (CuO) center can promote the formation of radical groups, and the solvent plays a vital role in controlling their formation (Figure 7). The improvement brought by P-doping might be related to improved charge storage and to the promotion of the electron-transfer mechanism, as suggested by the electrochemical analysis.

**Figure 7:**
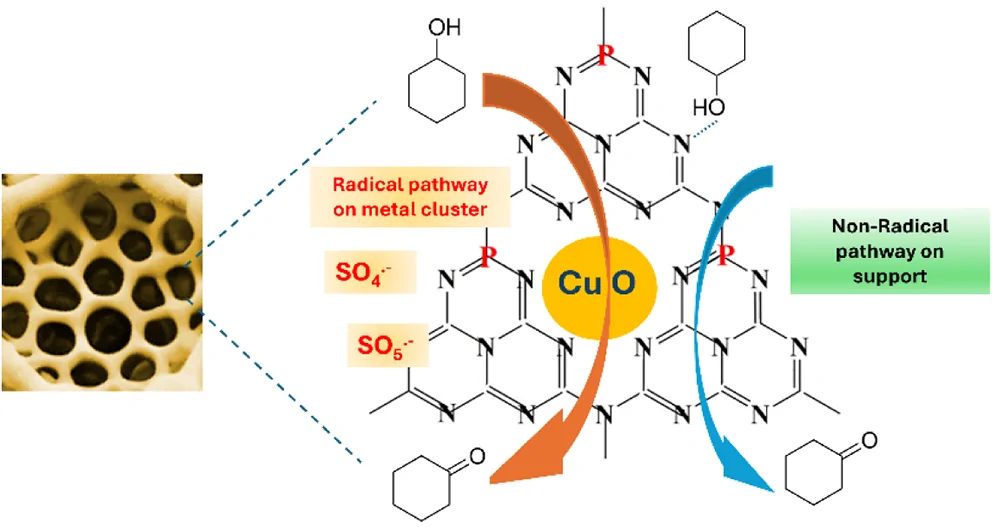
Proposed mechanism of PMS activation on the selective oxidation of cyclohexanol.

The recyclability of the MCF-CN–P-Cu and MCF-Cu catalysts was also investigated (Figure 6D), moreover, MCF-CN-Cu is also displayed in Figure S16. The MCF-CN–P-Cu can be recycled six times without significant loss of performance. The resulting liquid from the catalytic test was analyzed by ICP-OES, revealing no presence of copper. XRD was used to examine the spent catalyst for evidence of large metal particle formation or changes in the crystalline structure; however, no new peaks were observed in the diffractogram (Figure S17). On the other hand, the MCF-Cu undergoes rapid deactivation, and the XRD of the spent catalysts shows much more intense CuO peaks. This particle growth might be related to Ostwald ripening, in which smaller particles dissolve and reprecipitate onto larger ones.

## Experimental Section

### Chemicals

Pluronic P123 (Poly­(ethylene glycol)- block -poly­(propylene glycol)- block -poly­(ethylene glycol), tetraethyl orthosilicate (TEOS) ≥ 99.0%, ammonium fluoride ≥ 98.0%, potassium peroxymonosulfate, sodium azide (99.5%), and 4-hydroxy-3,5-dimethoxybenzyl alcohol were purchased from Sigma-Aldrich. Copper (II) nitrate trihydrate (99%) was purchased from Acros Organics. Mesitylene ≥ 98.0% was obtained from Merck. Acetonitrile (99.5%), acetone(99%), Dimethylformamide (99%), *n*-hexane (99%), toluene (98%), methanol(99.8%), ethanol (99.8%), tert-butanol (99%), and sodium hypophosphite monohydrate pure p.a. were purchased from ChemPur. Benzyl alcohol (99.5%), HCl solution 35–38%, and cyclohexanol were purchased from POCH S. A.

### MCF Synthesis

The mesoporous silica was synthesized using a methodology previously reported elsewhere.^97^

### MCF-CN Synthesis

The synthesis of carbon nitride layers on the MCF surface was performed using cyanamide as the chemical precursor. First, 600 mg of silica was dried under vacuum at 120 °C. Then, 300 mg of cyanamide was dissolved in 5 mL of ethanol, and the solution was added to the dried silica; the solvent was removed at 45 °C using a rotary evaporator. The obtained white solid was placed in a tubular oven (the calcination boat was covered) and subjected to thermal treatment at 550 °C for 4 h at a ramp rate of 3 °C min_–1_ under an argon atmosphere.

### MCF-CN-P Synthesis

P-doped carbon nitride was prepared using cyanamide and sodium hypophosphite as the doping precursor. In this procedure, 600 mg of MCF silica was dried under vacuum at 120 °C. Then, 300 mg of cyanamide and 72 mg of NaH_2_PO_2_*H_2_O were dissolved in 5.0 mL of ethanol. This solution was added to the dry MCF, and the solvent was removed under vacuum at 45 °C. The obtained white solid was placed in a tubular oven (the calcination boat was covered) and subjected to thermal treatment at 550 °C for 4 h at a ramp rate of 3 °C min^–1^ under an argon atmosphere.

### Copper Oxide Nanoparticle Confinement

CuNO_3_*3H_2_O (86 mg) was dissolved in 0.8 mL of distilled water. The solution was added dropwise to 500 mg of the desired support (MCF, MCF-CN, or MCF-CN-P). The obtained wet solid was dried under vacuum (60 °C) and subjected to thermal treatment at 400 °C for 2 h with a ramp rate of 3 °C min^–1^. The solids obtained were named MCF-Cu, MCF-CN-Cu, and MCF-CN–P-Cu.

### Catalyst Test

0.5 mmol of the desired alcohol and 0.55 mmol of PMS were added to 5 mL of the selected solvent. This solution was added to a vial containing 15 mg of the catalyst. This mixture was magnetically stirred for the specified time at the specified temperature. After the reaction cooled, the solid and liquid phases were separated by centrifugation. GC-FID was used to analyze the liquid, and the products were identified by GC-MS or by injection of a standard.

## Conclusions

Layers of carbon nitride or P-doped carbon nitride were successfully synthesized on MCF’s surface. The CuO nanoparticles were confined in these porous structures. In the pristine silica, CuO has a much larger particle size. The introduction of (P-)carbon nitride on the silica surface drove the formation of much smaller CuO nanoparticles. N- or P-species in the composites MCF-CN and MCF-CN-P are essential for coordinating the Cu-species and controlling their size. The three solids, MCF-Cu, MCF-CN-Cu, and MCF-CN–P-Cu, were used as catalysts for the selective oxidation of alcohols via PMS activation. The MCF-CN–P-Cu exhibits the best catalytic activity for the selective oxidation of cyclohexanol; moreover, both activity and selectivity are strongly influenced by the solvent. The quenching tests suggest two mechanisms operating in the oxidation reaction: the metal center (CuO) is responsible for the formation of radical species, which account for 15–20% of the cyclohexanol conversion, and the other path involves a nonradical mechanism. The electrochemical analyses suggested an electron-transfer mechanism, with P-doped carbon nitride showing the best performance.

## Supplementary Material



## Notes

TEM images of supports (Figure S1); TG analysis (Figure S2); XRD of graphitic carbon nitride (Figure S3); FTIR (Figure S4) and UV–vis (Figure S5) of the supports; SEM analyses of the composites (Figure S6); XPS analyses (Figure S7 and S8); XRD pattern of MCF-Cu (Figure S9); TEM images of MCF-CN-Cu and MCF-Cu (Figure S10 and S11); complementary catalyst tests (Figure S12, Figure S13, Figure S14); Linear sweep voltammograms (Figure S15); Recyclability of MCF-Cu catalyst (Figure S16) and XRD of fresh and spent catalysts (Figure S17). Textural properties of the composites (Table S1); C-bond distribution XPS C 1s high-resolution (Table S2); N-bond distribution XPS N 1s high-resolution (Table S3); yield of selective oxidation of cyclohexanol (Table S4); Comparison of Cu-based catalysts (Table S5) (PDF)
