# Supplementary material for: Copper Nanoparticle Confinement in P‑Doped Carbon Nitride Templates Enables Efficient Peroxymonosulfate Activation for Alcohol Oxidation
Source: ACS Omega. 2026 Aug 3;11(32):48563–73. doi: 10.1021/acsomega.6c05865 (PMC13491518; doi:10.1021/acsomega.6c05865)
Supplement: Supplementary file 1 [file ao6c05865_si_001.pdf]

Supporting Information

Copper Nanoparticle Confinement in P-Doped Carbon Nitride Templates Enables Efficient Peroxymonosulfate Activation for Alcohol Oxidation

Rafael L. Oliveira[a]*, Emilia Witkowska-Nery[b], Sebastian Praetz[c], Christopher Schlesiger[c]

[a] Institute of Low Temperature and Structure Research of the Polish Academy of Sciences, 50422, Wroclaw, Poland
[b] Institute of Physical Chemistry of the Polish Academy of Sciences, 01-224 Warsaw, Poland
[c] Institute of Physics and Astronomy, Technical University Berlin, 10623 Berlin, Germany

*r.oliveira@intibs.pl

Number of pages: 11

Number of Figures:17

Number of Tables: 5

Equipment

Thermo Fisher ScientificESCALAB 250Xi was used for X-ray photoelectron spectroscopy (XPS). The size of the X-ray spot on the sample is 400 µm, 20 scans for the survey, and 50 scans for regions. For each sample, a survey and high-resolution C1s, O1s, N1s, P2p, and Cu2p regions were measured. The Avantage software with pseudo-Voigt Gaussian−Lorentzian product functions and Smart background was used for peak deconvolution.

An X'Pert PRO PANalytical powder diffractometer, Cu Kα radiation, was used to study the crystalline structure of the composite materials. The X'Pert HighScore Plus program was used to display and analyze the diffraction patterns. Data were collected in the range of 2θ = 10–80˚. Derivatograph TG-DTA, MOM Budapest, was used to perform the thermogravimetric analysis (TGA). The samples (∼50 mg) were heated from room temperature to 800 °C with a rate of 5 °C/min under air and kept at 800 °C for 1 hour.

The N_2 physisorption isotherms were obtained at -196.15 ºC using a Micrometrics ASAP 2020 automated system. All carbonaceous materials were dried at 150 °C for 6 h under a vacuum before the measurement.

FEI Nova NanoSEM 230 microscope was used to collect the morphology of MCF-based materials. The chemical mapping was obtained from another microscope, a Tescan Mira3 SEM. The microstructure of the samples was investigated with a Philips CM-20 SuperTwin TEM microscope, operating at 160 kV. EDS chemical mapping images were obtained from a FEI Tecnai G2 20 STWIN microscope at an operating voltage of 200 kV.

The mid-IR ATR spectra were measured in the range of 4000–400 cm^{-1} using a Nicolet iS50 (Thermo Scientific) infrared spectrometer equipped with a diamond crystal as a reflection element. The spectral resolution was set to 4 cm^{-1}.

The laboratory XAS-spectrometer used in this work is a self-developed wavelength-dispersive spectrometer in von Hámos geometry.[1,2] The spectrometer is equipped with a micro focus X-ray tube, a curved, highly annealed pyrolytic graphite mosaic crystal, and a hybrid photon counting CMOS detector (*Dectris EIGER R 500K*) with 512 × 1030 pixels and a pixel size of 75 µm × 75 µm. The X-ray tube is optimised to reach 30 watts at 15 kV. For the Cu-K edge measurement, the tube was operated with a voltage of 17 kV and a current of 1450 µA. The beam path between the components was evacuated with vacuum tubes with 25 µm Kapton windows to lower the absorption in air and increase the efficiency of the spectrometer. To calibrate the energy of the spectrum, an 8µm copper foil was measured. To measure XANES with a high resolving power and to measure EXAFS in an adequate time, two different HAPG optics have been used.

For the XANES measurement, a 20 µm HAPG crystal with a bending radius of 300 mm has been used with an energy bandwidth of about 300 eV and a resolving power of E/ΔE = 4000. For the EXAFS measurement, a so-called "multipurpose optic" was used, which is 40 µm-thick HAPG crystal with a bending radius of 150 mm. With an energy resolving power of E/ΔE = 2000, it can measure an energy bandwidth of approximately 600 eV. [1]

The sample was prepared by mixing the raw material with the binder Hoechst wax C in a ratio of 1: 0.5 and grinding them with a mortar and pestle. The mixture was then pressed with a hydraulic press into a pellet with a 13mm diameter. The reference powders CuO and CuO_2 were prepared using the same method, with a 1:6 ratio of reference material to binder. The measurement time of the samples for EXAFS measurement was 10 h, and for the XANES measurement 5 h.

Normalization and FT of the EXAFS spectra were done using *ATHENA* of the *Demeter* software package.[3] When comparing the MCF-sample spectra with the references measured alongside, the highest agreement is with CuO (see normalized spectra-EXAFS and R-FT-EXAFS). This is supported by the FT-EXAFS (see R-FT-EXAFS) when comparing the peak position of the first and second shell peaks. The FT

of the EXAFS spectra was done in a thin range from 3 to 8 with dk of 1 and a Hanning window, and shows very similar results for the peak position of the MCF samples with a slight shift towards lower R values compared to CuO.

Gas chromatography was performed using a Hewlett-Packard HP6890 Series equipped with a 30 m capillary column containing (5%-phenyl)-methylpolysiloxane as the stationary phase using the following parameters: initial temperature 30 °C, temperature ramp 5ºC.min^{-1}, final 200 °C, and injection volume 0.2 µL. GC-MS analyses were performed on the Agilent 7890 A gas chromatography coupled with the Agilent 5975 C mass spectrometer equipped with an EI (electron ionization) source and a single quadrupole mass analyzer. Separation was performed on the Agilent HP-5 ms UI column (30 m ×250 µm×0.25 µm). The temperature program was 40°C for 5 min, then 10 °C/min to 320 °C, and held for 5 min. The temperature of the injector and the transfer line was 250 °C. Helium was used as a carrier gas at a flow rate of 1 mL/min in the constant flow mode. The samples were injected in the split mode (split ratio 1 : 20), and the injection volume was 0.2 µl. Mass spectra were recorded in the scan mode (33–500 m/z range). The identification of compounds was based on the comparison with the NIST Mass Spectral Program.

For the electrochemical analyses, a suspension of 10 mg/mL of the test compound in acetonitrile was prepared and mixed in an ultrasonic bath. After further vortexing, 100µL of the suspension was added to 200µL of Nafion® alcohol solution and vortexed again. Two layers of 2.5µL suspension were applied to the surface of 3mm diameter glassy carbon electrodes polished using 1 and 0.05µm alumina particles and sonicated in water.

Cyclic voltammetry was performed with a scan rate of 20mV/s with a step of 1 or 2 mV depending on noise level, vs Ag/AgCl 1M KCl reference electrode, the working electrode was glassy carbon 3mm, the counter electrode was platinum wire. All measurements were made in acetonitrile with 10mmol PMS with or without the addition of 2.5mmol benzyl alcohol or 2.5mmol cyclohexanol. The measurement cell was deoxygenated with argon. All results shown are after stabilization of the signal, which was sometimes achieved after the first scan, but more often after the second or third. All measurements were performed on Palmsens 4 potentiostat, using PStrace software.

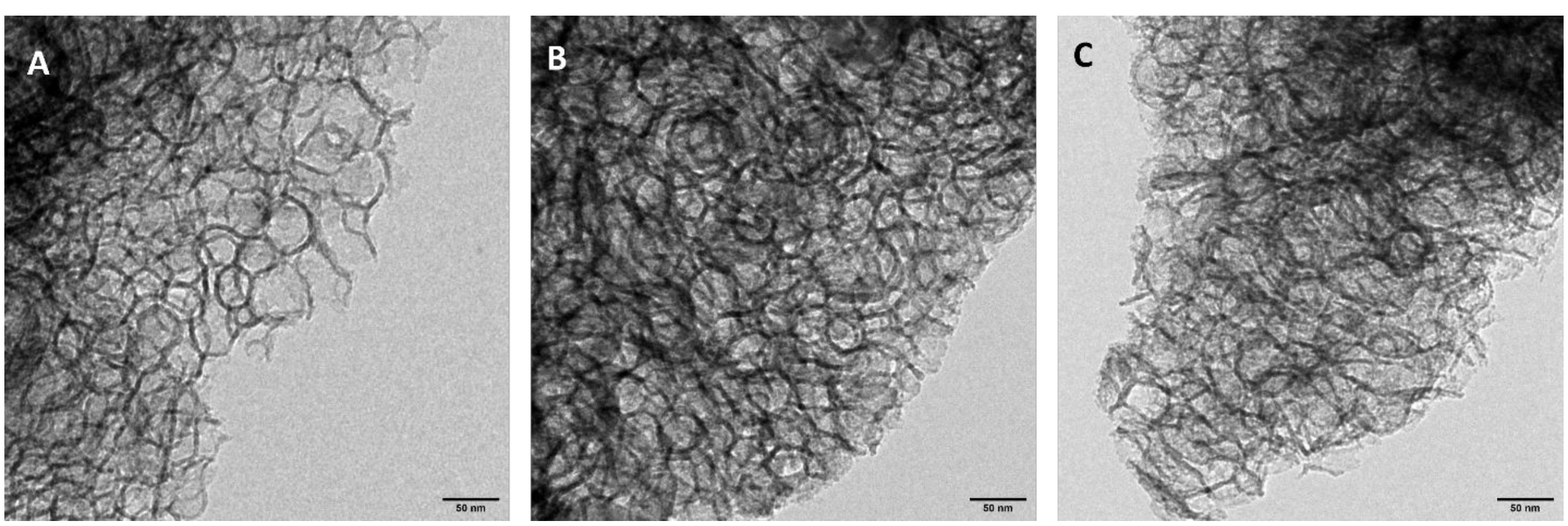

Figure S1 TEM of (A)MCF silica, (B)MCF-CN, and (C) MCF-CN-P, scale bar: 50 nm

Table S1 Textural properties of the synthesized composites

Material	*Surface area (m^2/g)*	*Pore volume (cm^3/g)*	*Percentage of organic matter*
MCF	628	2.05	-
MCF-CN	294	1.41	26
MCF-CN-P	290	1.4	23

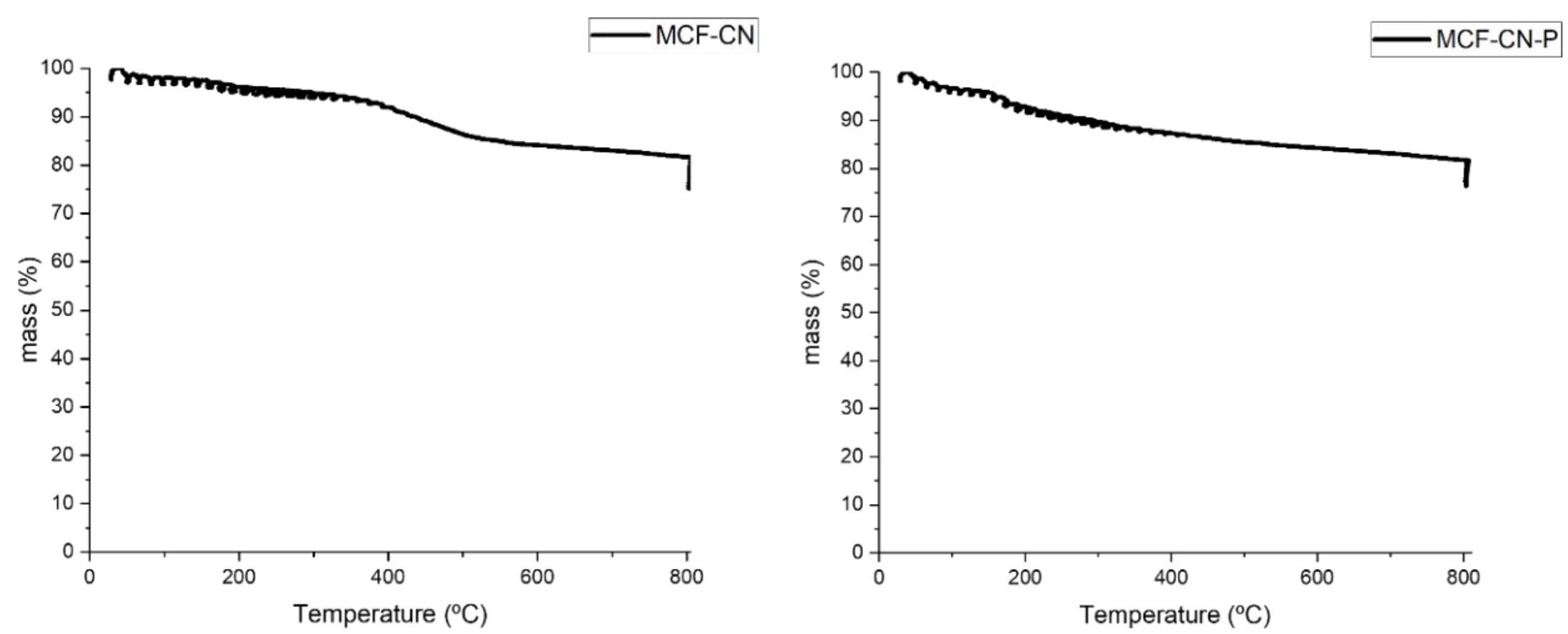

Figure S2 TG analysis of MCF-CN (left) and MCF-CN-P (right)

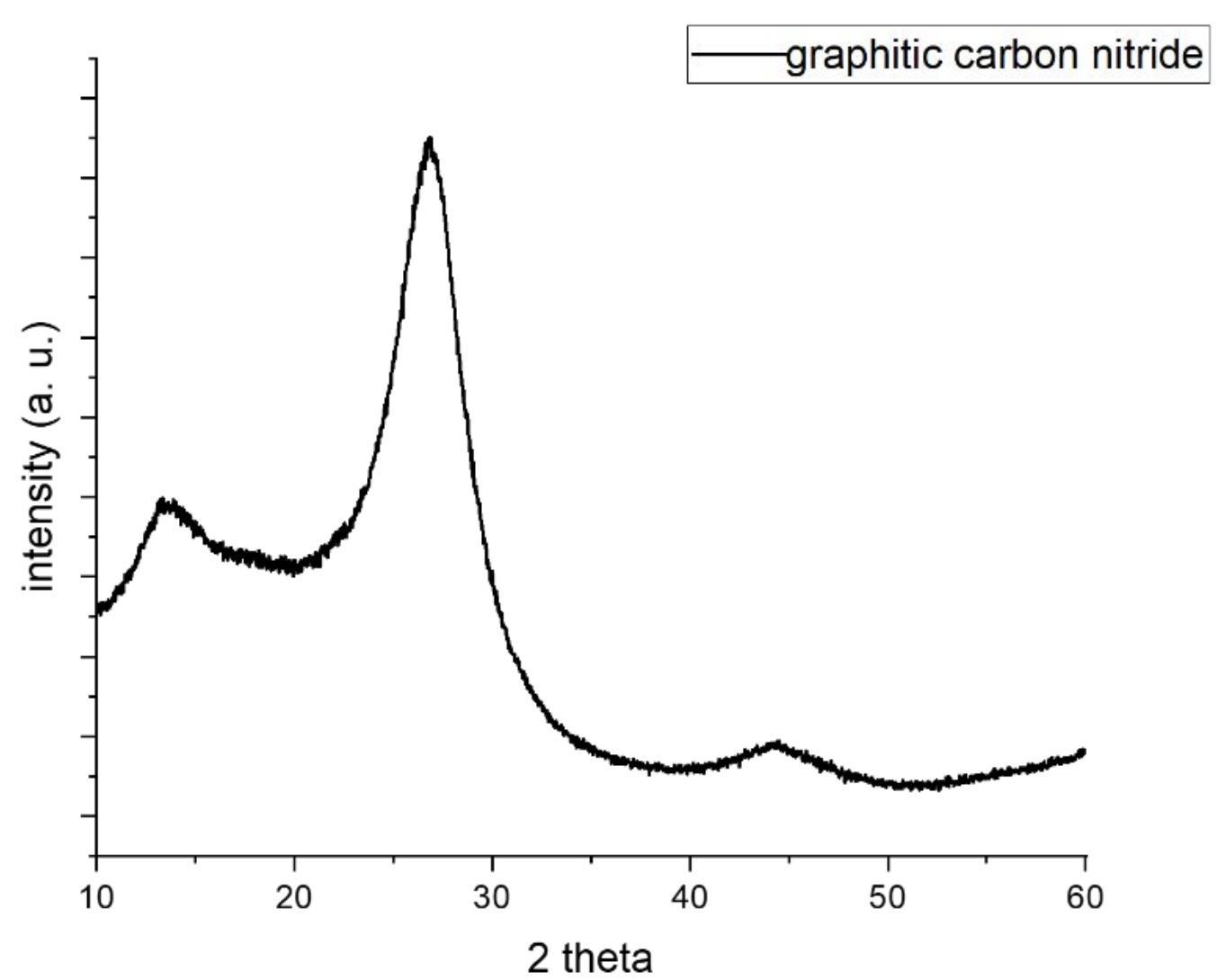

Figure S3 XRD pattern of graphitic carbon nitride

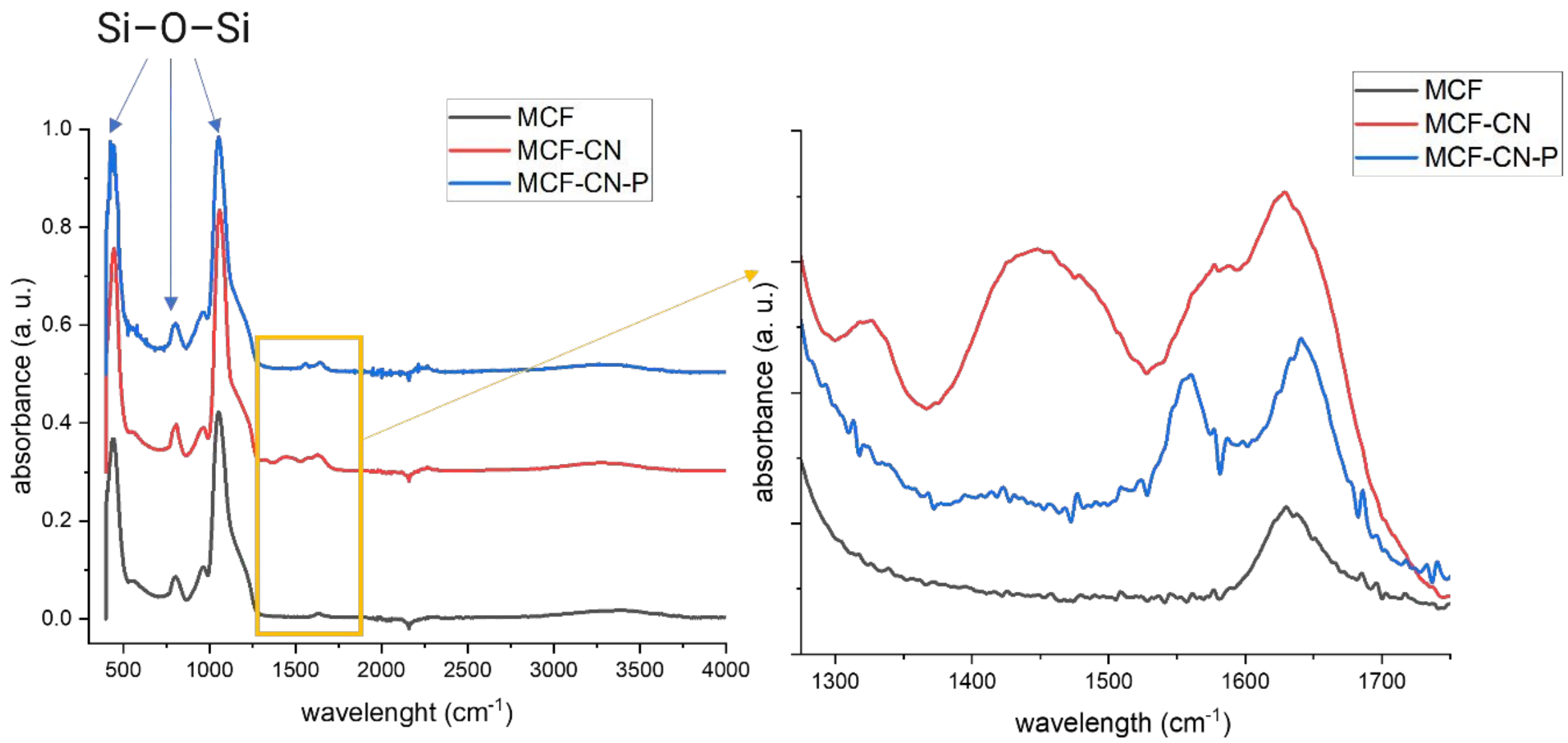

Figure S4 FTIR of MCF silica and the composites

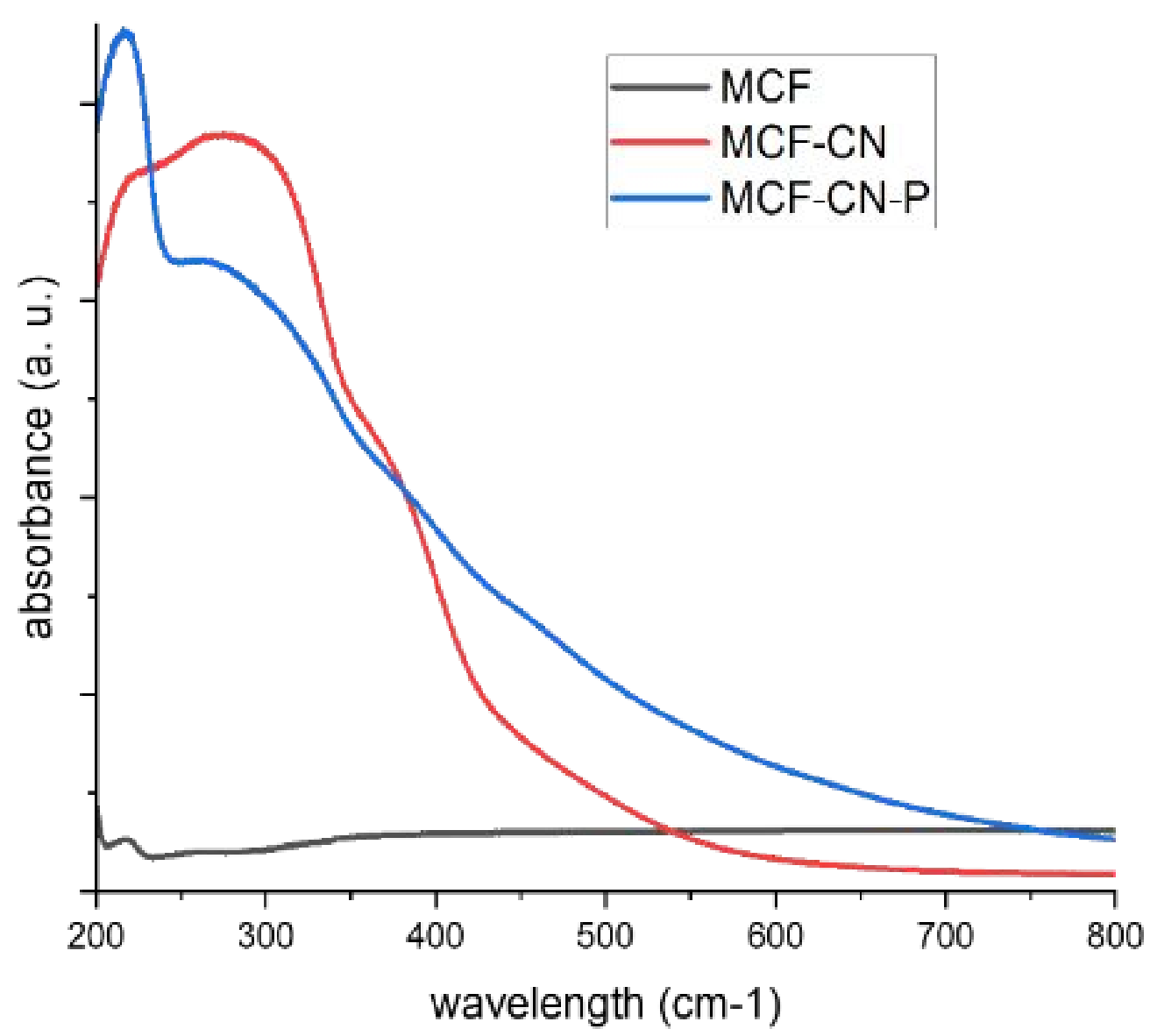

Figure S5 UV-vis of MCF silica and the composites

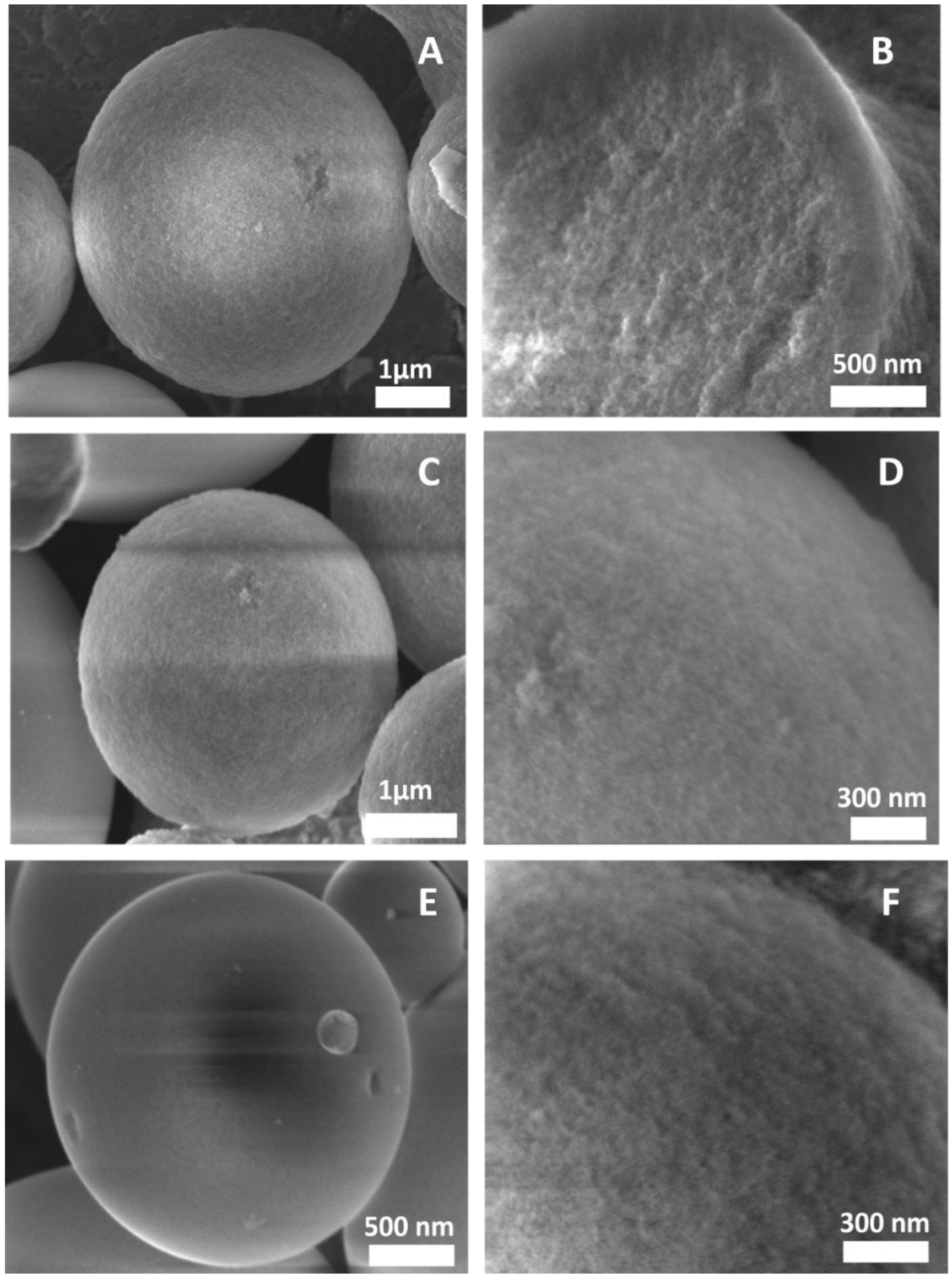

Figure S6 SEM images (A, B) pristine MCF silica; (C, D) MCF-CN, (E, F) MCF-CN-P

Table S2 C-bond distribution derived from XPS C 1s high-resolution spectra

MATERIAL	C-C	C≡C	C-NH_2	N-C=N	C-O
**MCF-CN**	34.3%	19.8%	8.3%	21.8%	15.8%
**MCF-CN-P**	37.2%	4.4%	14.5%	21%	22.9%

Table S3 N-bond distribution derived from XPS N 1s high-resolution spectra

MATERIAL	C=N-C	N-$(C)_3$	$(C)_2$-NH
**MCF-CN**	16.1%	48.2%	35.7%
**MCF-CN-P**	25.4%	45.2%	29.4%

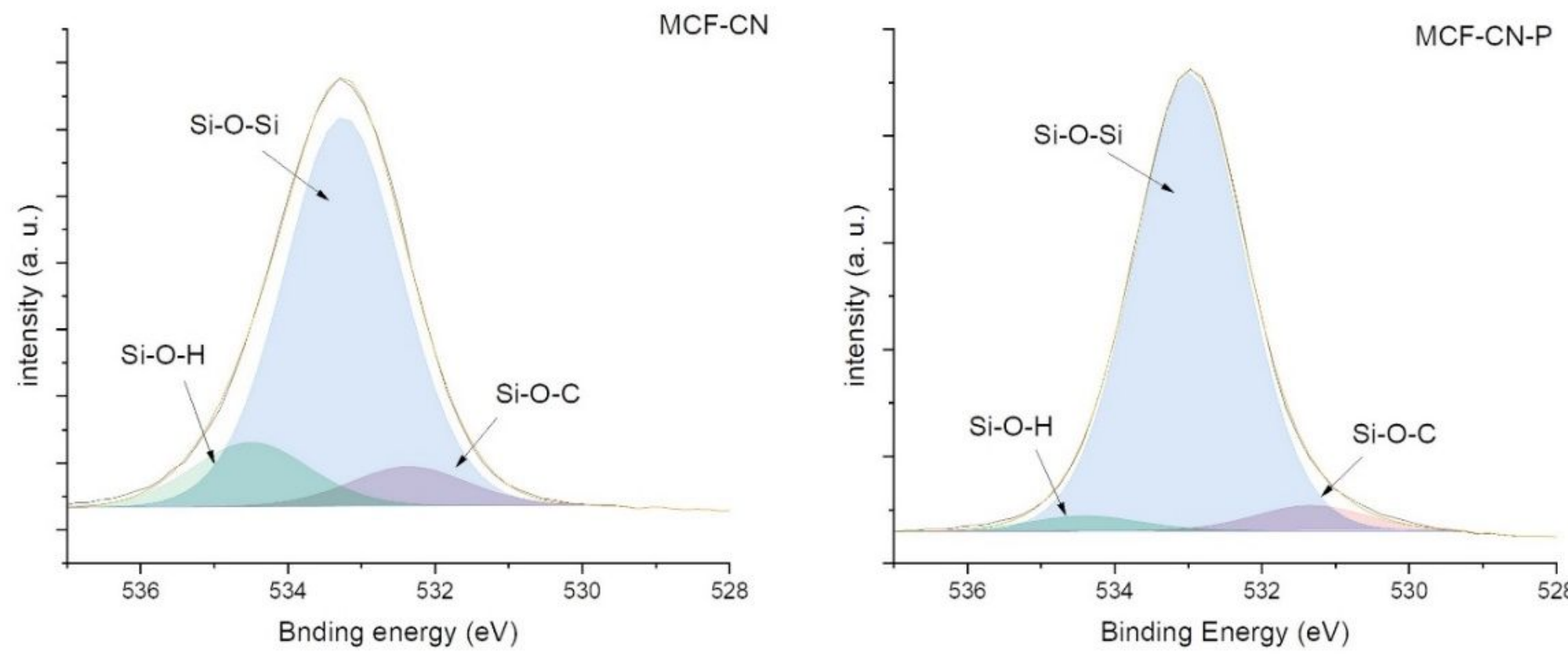

Figure S7 High-resolution O1s XPS spectra of MCF-CN and MCF-CN-P

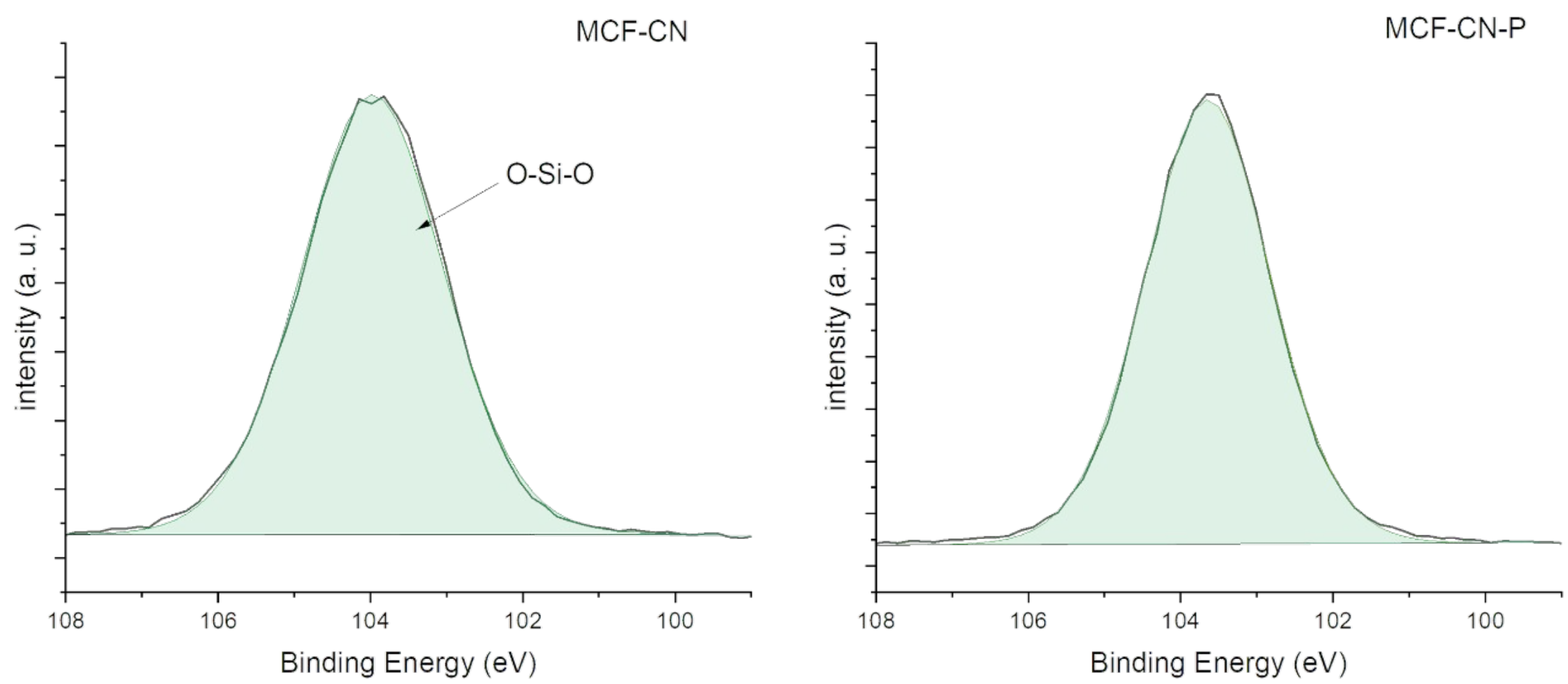

Figure S8 High-resolution Si2p XPS spectra of MCF-CN and MCF-CN-P

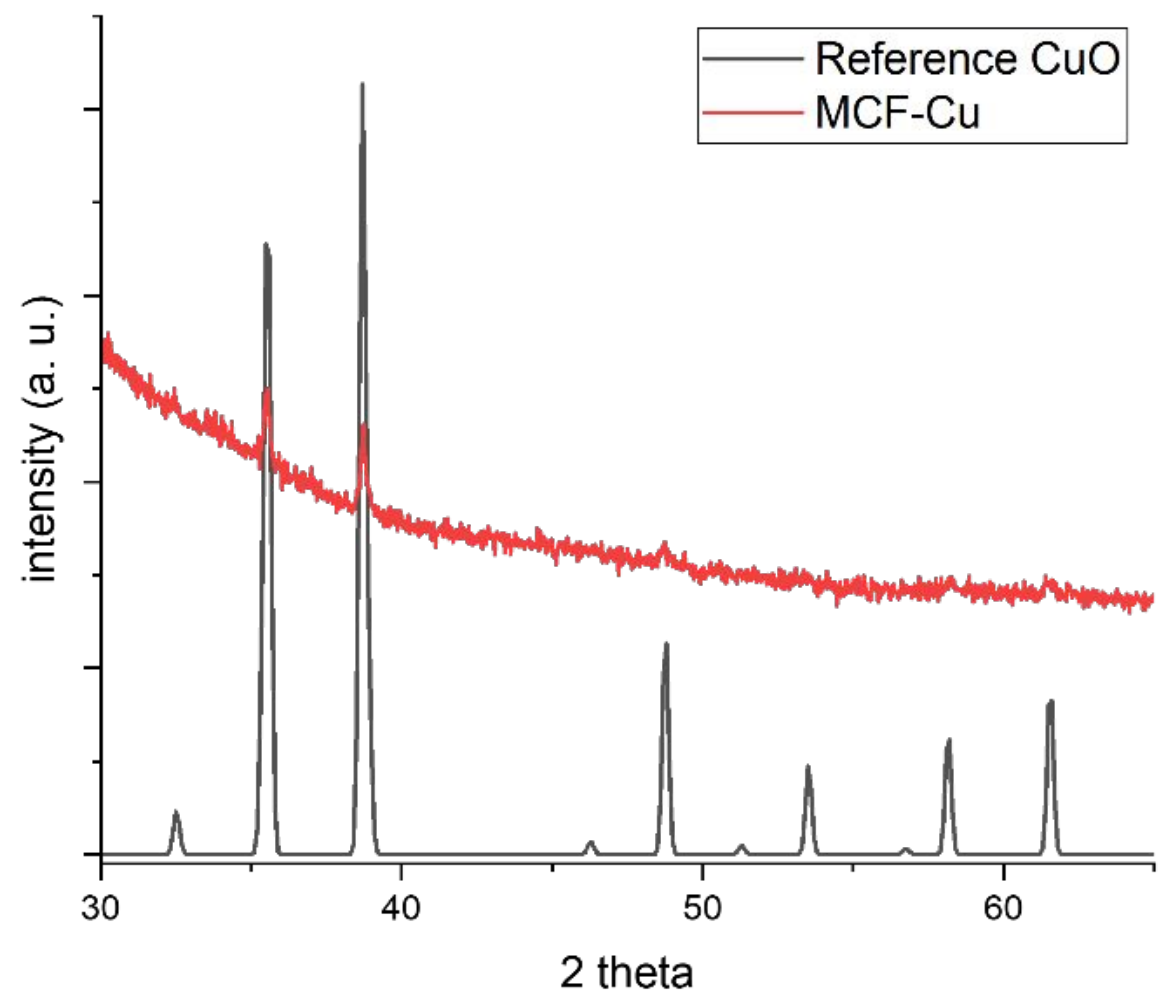

Figure S9 XRD pattern of MCF-Cu and the CuO monoclinic phase reference (Card #41-0254)

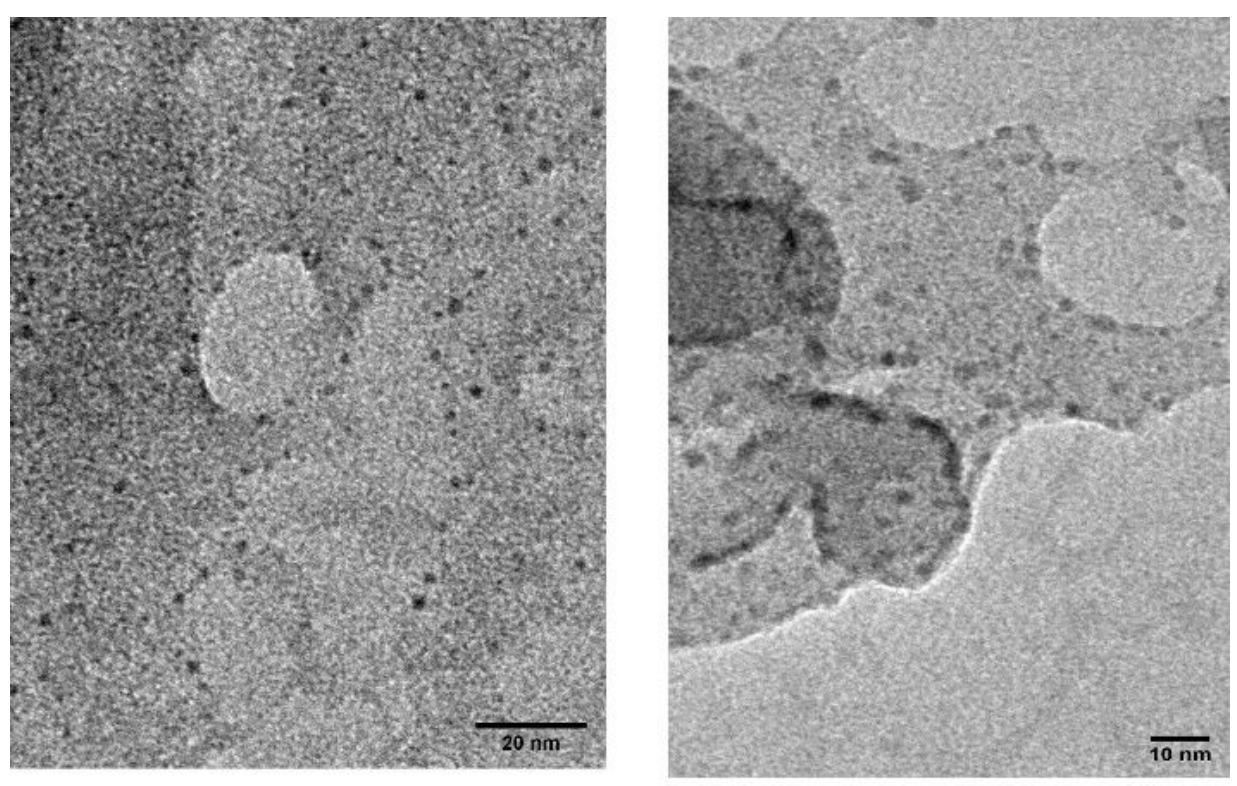

Figure S10 TEM images of MCF-CN-Cu

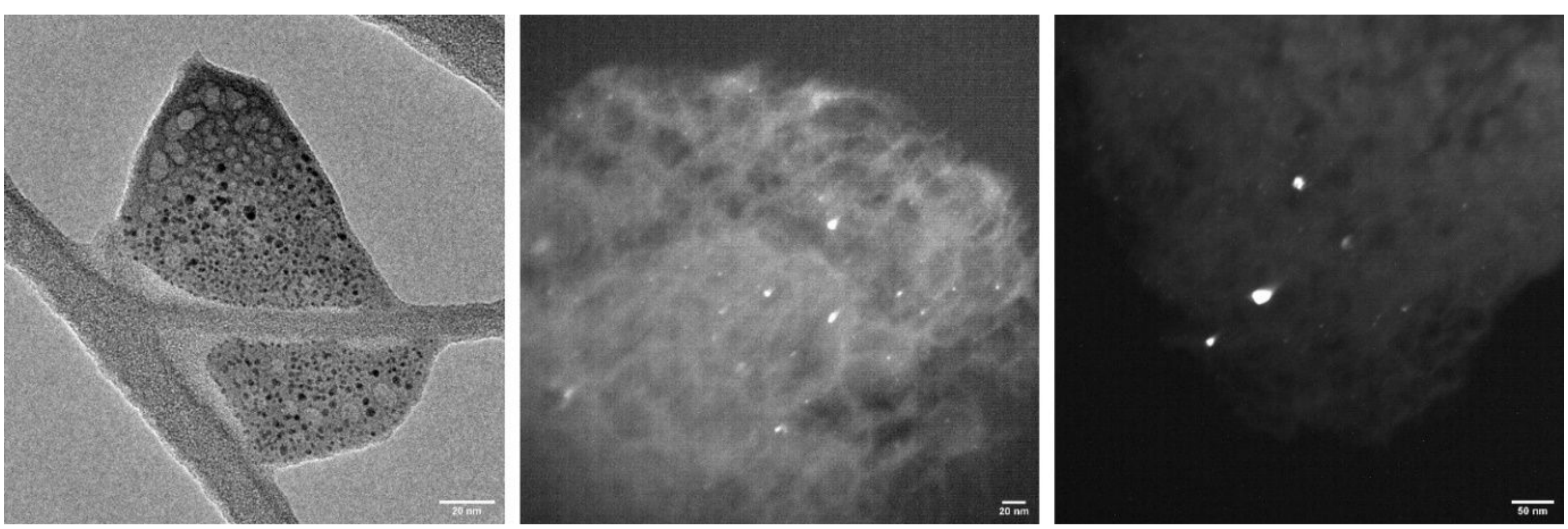

Figure S11 TEM (bright and dark field) images of MCF-Cu

Table S 4 Yield of selective oxidation of cyclohexanol derived from the results shown in Figure 5A

Catalyst	Yield (%)
MCF-Cu	43.73%
MCF-CN-Cu	74.03%
MCF-CN-P-Cu	84.33%

Conditions: 0.5 mmol of cyclohexanol, 0.55 mmol of PMS, 5 mL of acetonitrile, 15 mg of Cu-catalyst, 2 hours

$$\text{Yield (\%)} = (P_{CHO}/P_{CHOH}+P_{CHO}) \times 100$$

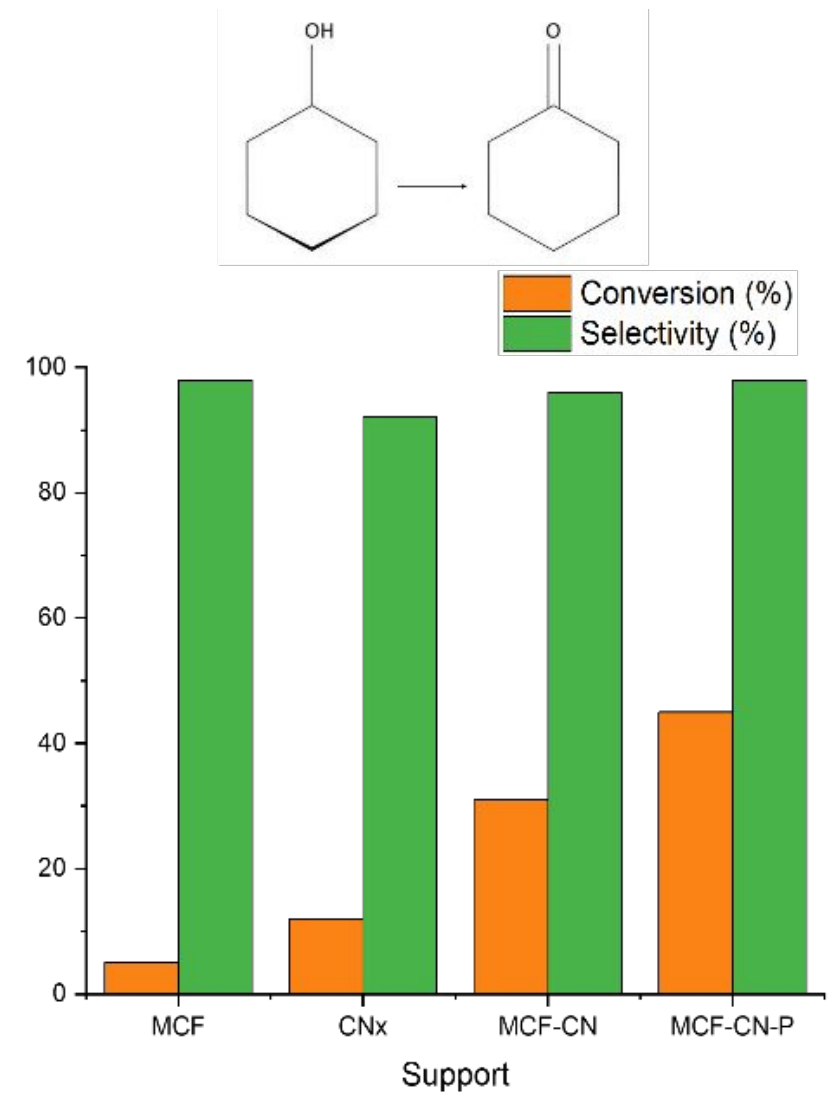

*Figure S12 The influence of the catalyst support on the selective oxidation of cyclohexanol. Conditions: 0.5 mmol of cyclohexanol, 0.55 mmol of PMS, 5 mL of acetonitrile, 15 mg of support, 2 hours, at 50ºC *CNx 4 mg (adjusted to the MCF-CN loading)*

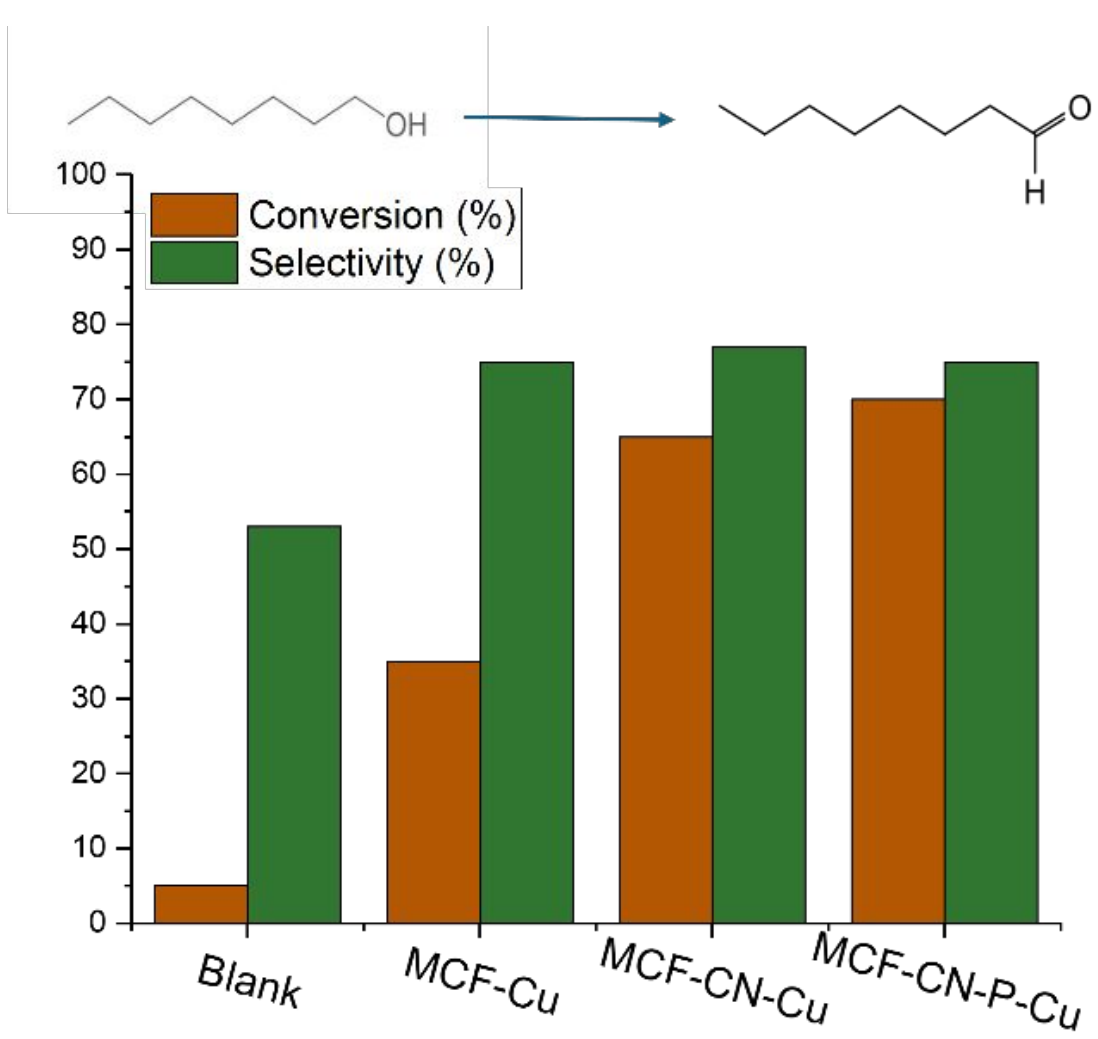

Figure S 13 The selective oxidation of 1-octanol. Conditions: 0.5 mmol of 1-octanol, 0.55 mmol of PMS, 5 mL of acetonitrile, 15 mg of support, 3 hours, at 50ºC

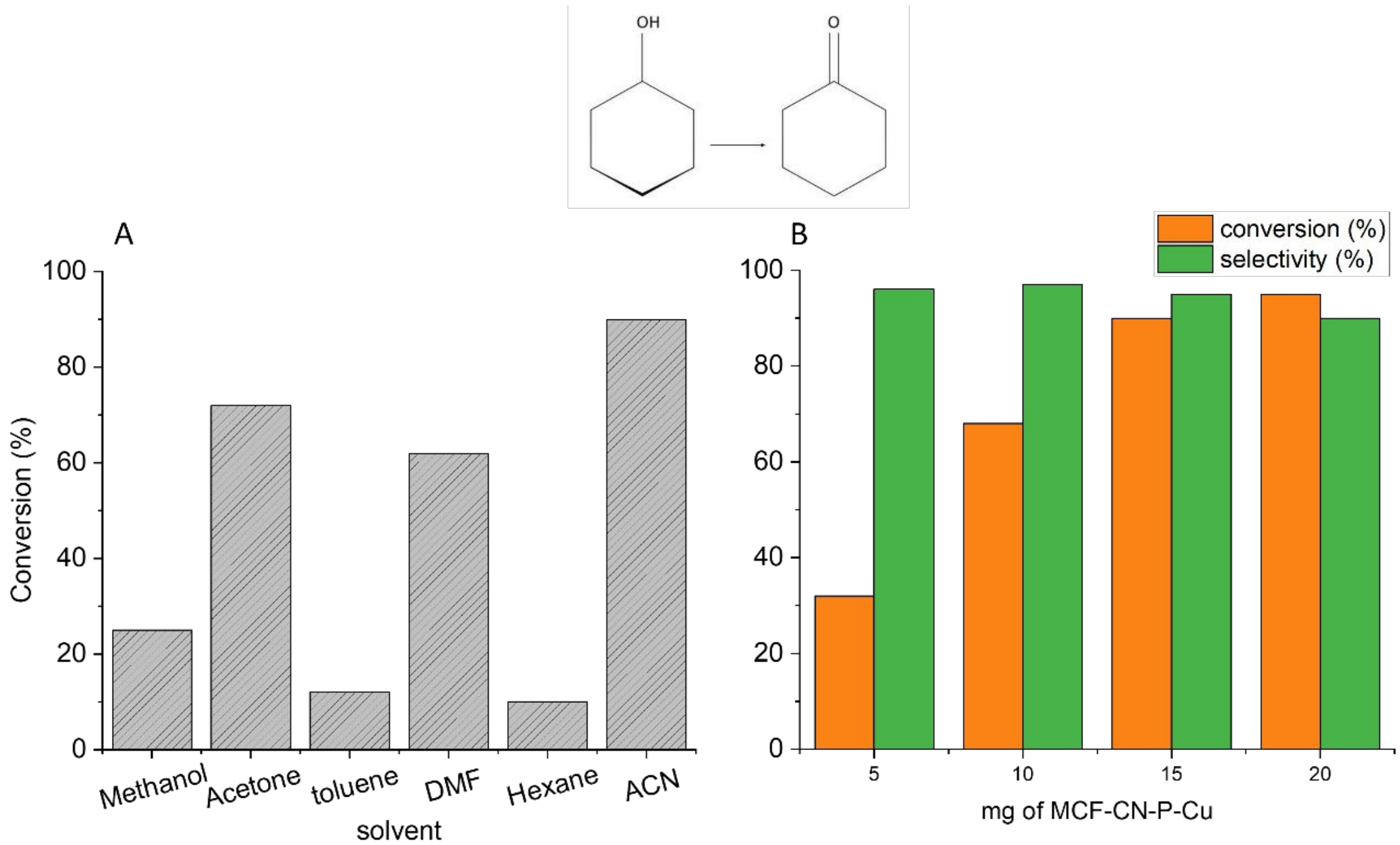

Figure S14 (A) The influence of the solvent on the selective oxidation of cyclohexanol. Conditions: 0.5 mmol of cyclohexanol, 0.55 mmol of PMS, 5 mL of solvent, 15 mg of MCF-CN-P-Cu, 2 hours, at 50ºC. (B) The influence of the MCF-CN-P-Cu loading on the selective oxidation of cyclohexanol. Conditions: 0.5 mmol of cyclohexanol, 0.55 mmol of PMS, 5 mL of solvent, X mg of MCF-CN-P-Cu, 2 hours, at 50ºC.

Table S5 Comparison of Cu-based catalysts to the material herein reported

References	Catalyst description and reaction conditions	TOF h^{-1*}
Reac. Chem. Eng. 2023, 8, 1061	Cu Oxide cluster deposited on N-doped carbon<br>45ºC, 2 hours, acetonitrile, peroxymonosulfate (PMS)	15.44
Tetrahedron, 2024, 151, 133769	Cu on Charcoal<br>80ºC, 24 hours, O_2 balloon, toluene	0.53
ACS Omega, 2024, 9, 970-976	Cu on Zeolite Y-type<br>60ºC, 6 hours, air	17
ChemNanoMat, 2018, 4, 71	Cu on pore glass<br>TEMPO , 100ºC, 4 hours, Toluene	5.22
Catal. Scie. Techn. 2022, 12, 1043	Cu on N-doped Carbon<br>60ºC, O_2 balloon, 20 h, TEMPO	4.95
**This work**	**Cu on P-doped Carbon nitride**<br>**50ºC, 2 hours, PMS, acetonitrile**	**23.6**

*TOF was calculated using the total loading of Cu deposited on the support

$$Cu^{2+} + HSO_5^{-} \longrightarrow Cu^{3+} + SO_4^{\cdot -} + HO^{-} \quad \text{Eq.1}$$

$$Cu^{3+} + HSO_5^{-} \longrightarrow Cu^{2+} + SO_5^{\cdot -} + H^{+} \quad \text{Eq.2}$$

$$Cu^{2+} + SO_4^{\cdot -} \longrightarrow SO_4^{2-} + Cu^{3+} \quad \text{Eq.3}$$

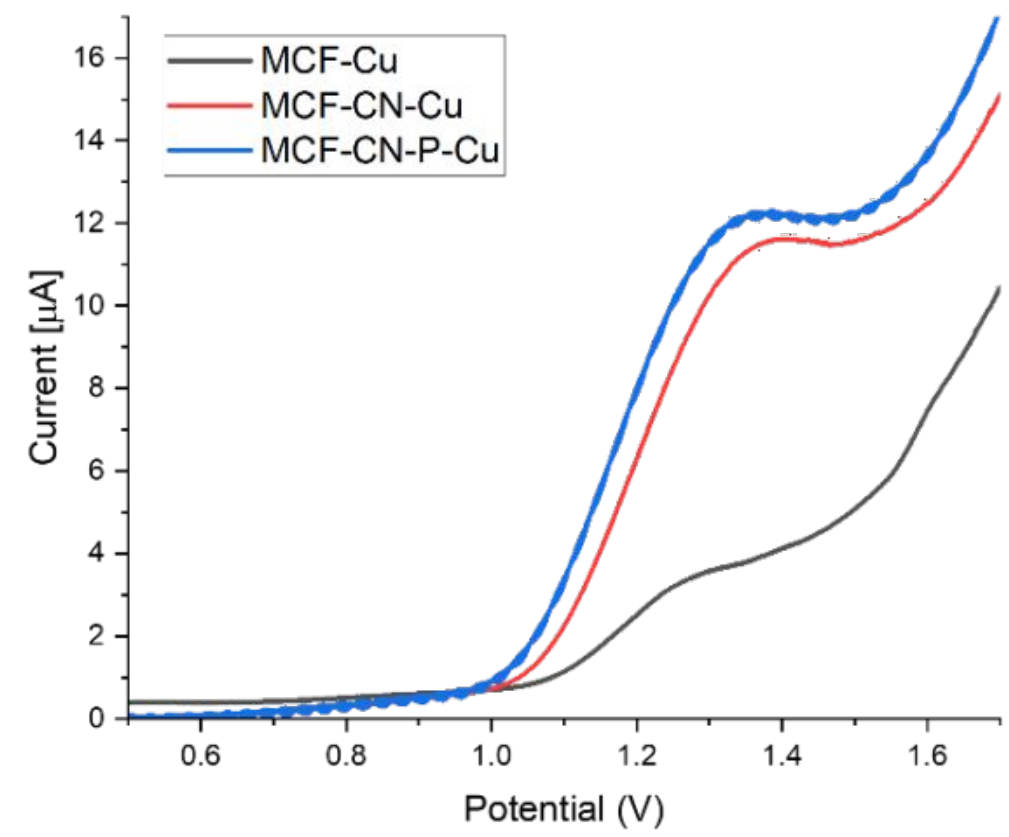

Figure S15 Linear-sweep voltammograms of the Cu-based catalysts in 10 mmol PMS with 2.5 mmol benzyl alcohol in acetonitrile, under argon, scan rate 20mV/s

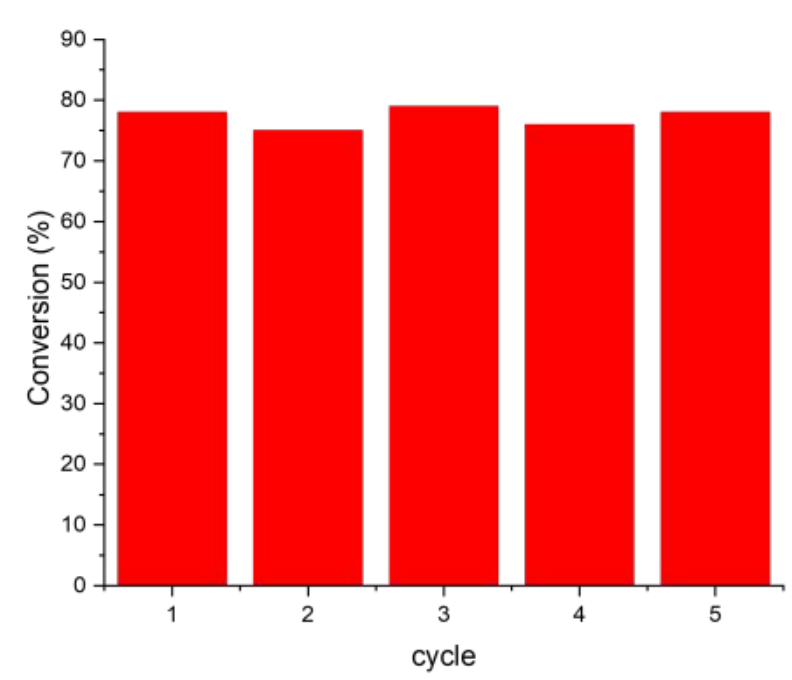

Figure S 16 Recyclability of MCF-CN-Cu in the selective oxidation of cyclohexanol.

conditions: 0.5 mmol of cyclohexanol, 0.55 mmol of PMS, 5 mL of solvent, 15 mg of MCF-CN-P-Cu, 2 hours, 50ºC

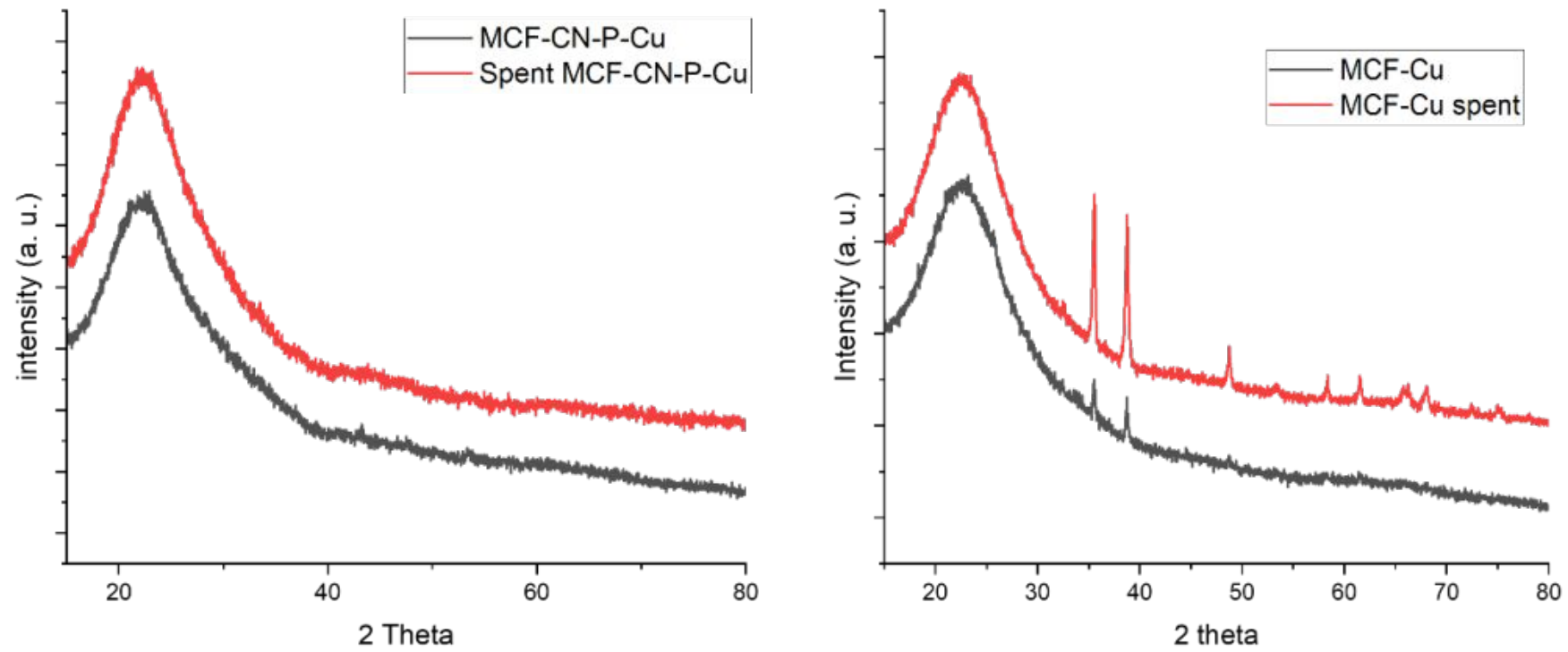

Figure S17 Fresh and spent catalyst after 6 cycles

[1] C. Schlesiger, L. Anklamm, H. Stiel, W. Malzer, B. Kanngießer, *J Anal At Spectrom* **2015**, *30*, 1080–1085.

[2] C. Schlesiger, S. Praetz, R. Gnewkow, W. Malzer, B. Kanngießer, *J Anal At Spectrom* **2020**, *35*, 2298–2304.

[3] B. Ravel, M. Newville, in *J Synchrotron Radiat*, **2005**, pp. 537–541.
